# All‐Amyloid Functional Coacervates

**DOI:** 10.1002/adma.74737

**Published:** 2026-08-24

**Authors:** Seyyed Alireza Hashemi, Mohammad Peydayesh, Laura Baraldi, Mohammad Arjmand, Raffaele Mezzenga

**Affiliations:** ^1^ Nanomaterials and Polymer Nanocomposites Laboratory School of Engineering University of British Columbia Kelowna BC Canada; ^2^ Department of Health Sciences and Technology ETH Zürich Zürich Switzerland; ^3^ Department of Materials ETH Zürich Zürich Switzerland

**Keywords:** amyloid nanofibrils, charge‐mediated complexation, functionalization‐assisted complexation, proteinaceous complex coacervates, soft matter

## Abstract

Electrostatic complex coacervates offer a robust pathway to convert homogeneous oppositely charged polyelectrolyte solutions into condensed structured liquids via liquid–liquid phase separation (LLPS). Directing these principles toward artificial, all‐proteinaceous complex coacervates enables the development of soft matter systems fully compatible with a broad range of advanced biotechnological products. Here, we employ charge‐tunable β‐lactoglobulin and lysozyme amyloid nanofibrils as building blocks toward fully proteinaceous functional materials. The functionality is dictated by engineering the composition and self‐organization of oppositely charged amyloid nanofibrils at the liquid–liquid interface. Depending on the path and process followed, we demonstrate all‐amyloid interfacial complex coacervates in the form of vein‐like tubular structures or capsules with tunable electrical or ionic conductivity, viscoelasticity, multidimensional morphology, and programmable macromolecular crowding. These constructs, made solely of amyloid constituents, provide a general platform for artificial protein scaffolds and advanced materials with potential applications in bioelectronics and bio‐optics.

## Graphical abstract

This study introduces an all‐amyloid interfacial complex coacervate technique that employs charge‐mediated or functionalization‐assisted complexation of oppositely charged food protein‐derived amyloid nanofibrils to engineer functional soft constructs across multiple length scales. This versatile strategy enables the fabrication of proteinaceous vein‐like tubules, bioelectronic and iontronic circuits, soft electromagnetic shields, viscoelastically tunable constructs, and bio‐optical systems.

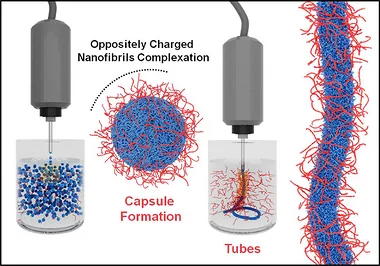

## 1 Introduction

Supramolecular self‐assembly and induced liquid–liquid phase separation (LLPS) represent complementary strategies for organizing soft matter into 3D‐structured, dynamic systems via non‐covalent interactions [1, 2]. They enable the formation of engineerable and tunable compartments through selective molecular organization and phase partitioning [3]. This provides a powerful framework for developing programmable, adaptive soft‐matter systems capable of controlling composition, functionality, reaction kinetics, and molecular transport [4, 5, 6]. These capabilities underpin their use in advanced applications, including targeted drug delivery and biomedical engineering [5], enzymatic microenvironments [6], and stimuli‐responsive functional materials [7].

Complex coacervation is an effective LLPS strategy that allows the formation of engineerable artificial proteinaceous compartments analogous to biomolecular condensates by mimicking their viscous and charged nature [8]. Their formation enables a wealth of applications in biotechnology and sustainable soft‐matter manufacturing [9, 10]. Coacervates are generally classified into simple and complex types. The classical definition of simple coacervates involves the separation of a uniform colloid dispersion into colloid‐rich (coacervate) and colloid‐poor (supernatant) phases within the same liquid phase. By contrast, complex coacervates involve a mixture of two oppositely charged polyelectrolytes that separate into concentrated and dilute phases in the same liquid, with both polyelectrolyte species present in each phase [9, 11, 12, 13]. Unlike conventional bulk coacervates, interfacial complex coacervation happens when oppositely charged polyelectrolyte dispersions are brought into contact via controlled extrusion. This confines electrostatic complexation and liquid–liquid phase separation to the contact boundary between the liquid phases, forming a dense interfacial complex coacervate layer [3].

Macromolecular complex coacervates are primarily composed of three main classes of material, including charged polysaccharides, synthetic polymers, and polypeptides. Physicochemical parameters such as pH, concentration, temperature, ionic strength, and charge density play a critical role in governing the formation of coacervates [4, 8]. Among macromolecular complex coacervates, the complexation of charged polypeptides with oppositely charged colloids provides a practical and simplified model for generating functional proteinaceous complex coacervates. Their interactions with other charged molecules can be tuned by varying the polypeptide sequence and side‐chain functionality [14]. However, the use of polypeptides is limited by their high molecular weight, complex composition, and demanding protein extraction and purification processes. This increases costs and limits scalability, posing a challenge for their deployment in real‐world applications [15, 16, 17]. Therefore, alternative charged proteinaceous colloids are needed that offer tunable physicochemical properties, structural robustness, simple processing, low cost, and scalability.

Among the most promising alternatives are amyloid nanofibrils derived from agricultural and food industry side streams or low‐cost protein sources such as whey [18, 19] and lysozyme [20, 21]. Amyloid nanofibrils are defined as supramolecular, high‐aspect‐ratio fibrillar assemblies formed by the misfolding and self‐assembly of polypeptide chains into elongated nanofibrils [22, 23]. They form from single or multiple filaments twisted together, yielding a fibrillar assembly with rigid or semi‐flexible character [24]. Their molecular structure arises from hydrogen‐bonded cross β‐strands that align perpendicular to the nanofibril axis [22]. Under optimized synthesis conditions, amyloid nanofibrils can be produced at scale from protein precursors derived from plant (e.g., oat [25] and soy [26]) and/or animal sources (e.g., lysozyme [27] and β‐lactoglobulin [28]). This makes amyloid nanofibrils a scalable and potentially cost‐effective source of precursors for artificial proteinaceous compartments.

Amyloid nanofibrils and polypeptides stand out as promising functional and adjustable proteinaceous nanofibrils with a wide range of potential applications across various disciplines. Examples include biosensing, drug release, and cell growth bio‐scaffold [29], engineered biomolecular condensates [30], sustainable food packaging [31, 32], chemical detoxification [28], artificial intelligence (AI) driven biomaterial discovery [33, 34], CO_2_ capture [35] and conversion [36], environmental remediation [37], effective food supplement delivery [25], wound treatment [38], antibacterial therapies [27], precious metals recovery [39], and beyond. They also exhibit tunable net surface charge via pH modulation and abundant surface functionalities [3, 40]. This enables their use as charge‐adjustable colloidal polyelectrolytes for engineerable soft materials through complexation with oppositely charged colloids, such as hyaluronic acid [3], phytic acid [41], and polysaccharide nanofibrils like cellulose nanofibril (CNF) and cellulose nanocrystal (CNC) [42]. However, a scalable strategy based solely on the complexation of oppositely charged amyloid nanofibrils to form fully proteinaceous interfacial complex coacervates remains lacking.

Herein, we present an all‐amyloid nanofibril interfacial complex coacervation strategy in which oppositely charged amyloid nanofibrils form soft, engineerable proteinaceous compartments through LLPS confined to the liquid–liquid interface. The interfacial complex coacervation can be driven by charge‐mediated or functionalization‐assisted complexation, using either the same or different amyloid nanofibril types, yielding robust, stable, and tunable constructs. We show that the engineerability of interfacial complex coacervates enables a wealth of functionalities, with examples in artificial proteinaceous veins, bioelectronics, iontronics, and bio‐optics.

## 2 Results and Discussions

### 2.1 Liquid‐in‐Liquid Structuring via Oppositely Charged All‐Amyloid Nanofibril Complexation

The amyloid nanofibrils were prepared from β‐lactoglobulin (pH 2.0, stirred at 90°C for 5 h) and lysozyme (pH 2.0, stirred at 90°C for 24 h) through unfolding and self‐assembly of hydrolyzed polypeptide chains (Figures 1A–C, S1–S3, and Experimental Section), following previously well‐established protocols [24, 43]. Adjusting the pH of amyloid nanofibrils from 2.0 to 12.0 tunes their net charge from positive to negative, thereby enabling the formation of all‐amyloid nanofibril proteinaceous compartments as capsules (Figure 1D) or non‐equilibrium‐shaped tubular interfacial complex coacervates (Figure 1E). These assemblies form when aqueous dispersions of oppositely charged amyloid nanofibrils are brought into contact by controlled extrusion, followed by interfacial complexation.

**FIGURE 1:**
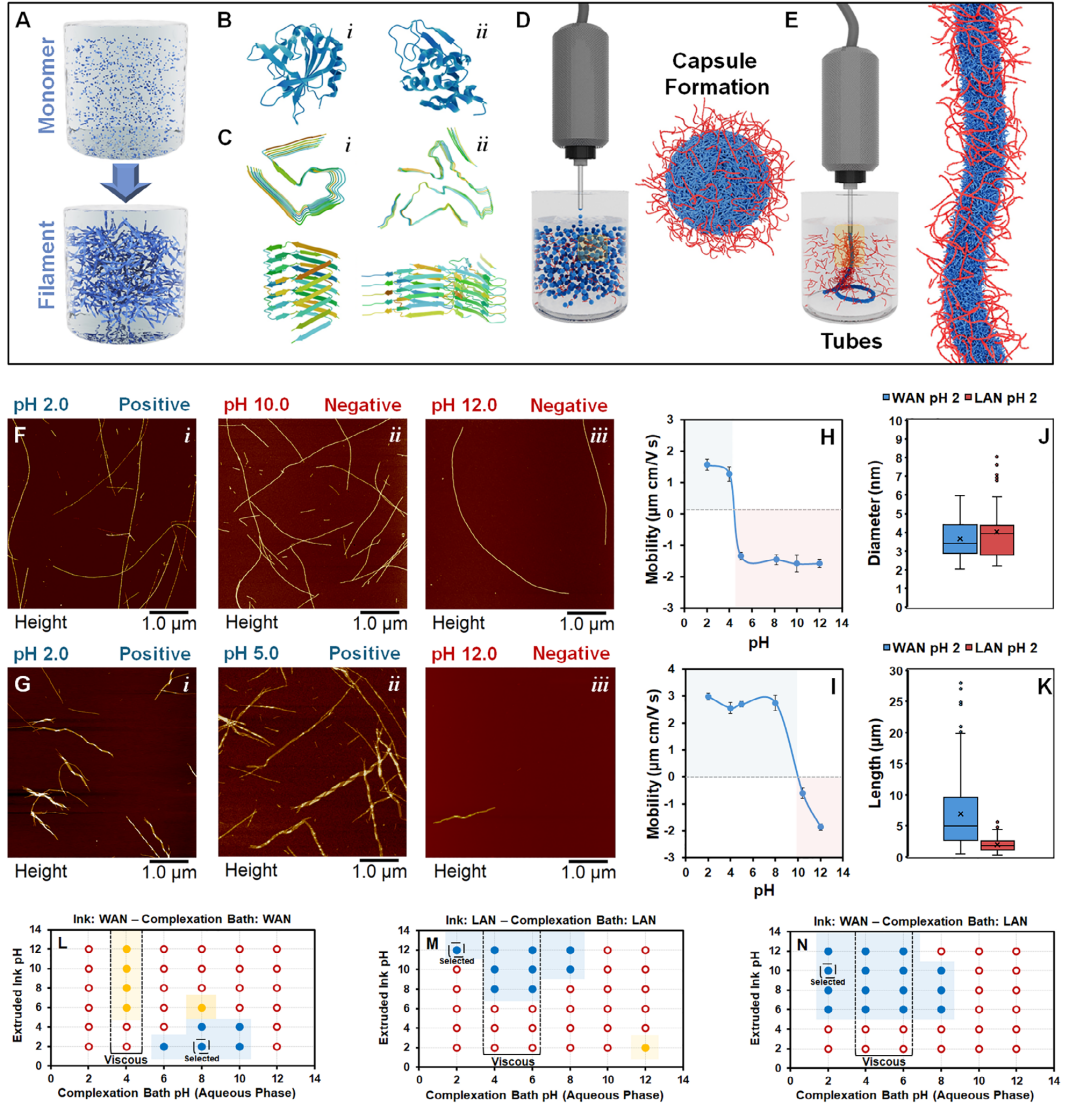
(A) Schematic illustration of WAN and LAN production upon adjusting the monomer mixture's pH to 2.0 and heating at 90°C for 5 and 24 h, respectively. 3D view of the (B) i: β‐lactoglobulin and ii: lysozyme protein monomers, and (C) when they turn into nanofibrils from cross‐section and longitudinal direction for i: β‐lactoglobulin and ii: lysozyme protein monomers. The graphic illustrations of the protein monomers and amyloid nanofibrils were created based on experimentally determined structures available in the Protein Data Bank (PDB), including PDB IDs 3NPO (β‐lactoglobulin), 7VGO (lysozyme), 9IAH (β‐lactoglobulin amyloid fibril), and 8QV8 (lysozyme amyloid fibril). Charge‐mediated complexation of oppositely charged amyloid nanofibrils during extrusion of an amyloid nanofibril ink into an oppositely charged amyloid nanofibril complexation bath, resulting in the formation of interfacial complex coacervate (D) capsules and (E) non‐equilibrium‐shaped tubes via dropwise or continuous extrusion, respectively; red and blue filaments represent oppositely charged amyloid nanofibrils. AFM images of (F) (i–iii) WAN and (G) LAN (i–iii) at various pH values. Electrophoretic mobility of (H) WAN and (I) LAN at various pH values (2–12), showing their either net positive or negative charges at each respective pH value. (J) Diameter and (K) length distribution ± standard deviation for WAN and LAN at pH 2.0. In parts (J) and (K), the cross indicates the mean value, the box shows the interquartile range (25%–75%), the whiskers indicate the data distribution excluding outliers (individual points) beyond the whisker limits, with standard deviation reported as an additional measure of variability. pH‐dependent complexation phase diagram when extruding (L) WAN into WAN, (M) LAN into LAN, and (N) WAN into LAN at various inks’ and complexation baths’ pH values; the concentration of amyloid nanofibrils dispersion was set 2 wt% and complexation bath (2 wt%) was mixed with 20 vol% isopropyl alcohol prior to ink extrusion; the complexes were heated at 80°C for 10 min after extrusion to improve their stability; the phase diagrams divided into three regions including red hollow circles (no capsule formed), yellow circles (capsule formed but difficult to shape), and blue circles (well‐defined capsule formation).

AFM morphological analysis of whey amyloid nanofibrils (WAN) (Figure 1Fi–iii) and lysozyme amyloid nanofibrils (LAN) (Figure 1Gi–iii) showcases their nanofibrillar morphology with a high aspect ratio that preserves their nanofibrillar arrangement over a wide range of pH values (2.0–12.0). Assessing the electrophoretic mobility of amyloid nanofibrils across a broad pH range reveals that WAN exhibits a broader negative‐charge window, with an isoelectric point around pH ∼4.4; whereas LAN displays a stronger and more persistent positive‐charge character, with an isoelectric point near pH ∼10 (Figure 1H–I). This charge‐complementarity allows precise control of electrostatic interactions, extends the pH range for forming proteinaceous compartments, and increases design flexibility.

The diameter (Figure 1J) and length (Figure 1K) distributions of WAN and LAN (pH 2.0, 0.01 wt%) were further examined by AFM analysis (large AFM scans shown in Figures S4 and S5). WAN and LAN exhibited mean diameters ± standard deviation of 3.64 ± 1.03 nm (range: 2.01–5.95 nm) and 4.02 ± 1.41 nm (range: 2.19–8.02 nm), respectively. These results are consistent with the formation of amyloid nanofibrils composed of single filaments or bundled/twisted assemblies, with LAN exhibiting a broader diameter distribution [24, 43]. Length distribution analysis (Figure 1K) revealed mean contour lengths ± standard deviation of 6.85 ± 1.92 and 5.69 ± 1.06 µm for WAN and LAN, respectively. Length distributions were right‐skewed, with a higher maximum contour length observed for WAN (∼27.98 µm) compared to LAN (∼5.80 µm). This reflects the intrinsically high aspect ratios of ∼1.88 × 10^3^ and ∼1.42 × 10^3^ for WAN and LAN, respectively (contour length over diameter (*L*_c_/*D*) considering mean values).

The amyloid nanofibrils were complexed into interfacial complex coacervate capsules over a wide pH range (2.0–12.0) to determine the optimal pH for their complexation. The complexation was monitored at various pH values upon extruding a droplet of 2 wt% either positively or negatively charged all‐aqueous amyloid nanofibril ink into the 2 wt% either positively or negatively charged amyloid nanofibril complexation bath pre‐mixed with 20 vol% isopropyl alcohol. The addition of alcohols (e.g., isopropyl alcohol) could promote LLPS by lowering the solvent dielectric constant and weakening nanofibril solvation (Figure S6), partially disrupting hydration shells and strengthening nanofibril‐nanofibril electrostatic interactions that drive complexation [44, 45, 46]. All interfacial complex coacervate capsules were heated at 80°C for 10 min after complexation to improve their long‐term stability. Without this post‐treatment, individual capsules gradually dissociated in the complexation bath and lost structural integrity over time (Figure S7). These results show that a simple heating step is sufficient to stabilize the as‐developed all‐amyloid structures.

Three types of all‐amyloid nanofibril complexation systems were investigated by extruding (i) WAN ink into the WAN complexation bath (Figures 1L and S8), (ii) LAN ink into the LAN complexation bath (Figures 1M and S9), and (iii) WAN ink into the LAN complexation bath (Figures 1N, S10, and S11); all at the fixed concentrations of 2 wt% but across varying pH values. The pH‐dependent complexation phase diagram is divided into three regions: no capsule formed (red hollow circles), uncontrolled‐shape capsule (yellow circles), and well‐defined‐shape capsule (blue circles) (Figure 1L–N). As depicted, the best pH value for each type of system was selected as extruding (i) WAN ink pH 2.0 into WAN complexation bath pH 8.0, (ii) LAN ink pH 12.0 into LAN complexation bath pH 2.0, and (iii) WAN ink pH 10.0 into LAN complexation bath pH 2.0. At these selected points, amyloid nanofibrils possess strongly enough net opposite charges at pH values well away from their isoelectric point (Figure 1H,I), forming stable interfacial complex coacervate capsules.

Microscopic visualization revealed that all three optimized formulations yielded a tubular morphology (Figure 2A–C); whereas WAN into WAN complexation exhibited limited control over the tubes' shape and morphology (Figure 2A and Video S1). On the other hand, for LAN into LAN (Figure 2B and Video S2) and WAN into LAN (Figure 2C and Video S3) systems, the tubes’ morphology can be optimized via controlled extrusion. The WAN into LAN all‐amyloid nanofibril complexation system was selected as the most optimized because it demonstrated better morphology control and complexation quality. This may result from stronger heterotypic electrostatic interactions between β‐lactoglobulin and lysozyme amyloid fibrils, which differ in surface chemistry and isoelectric point.

**FIGURE 2:**
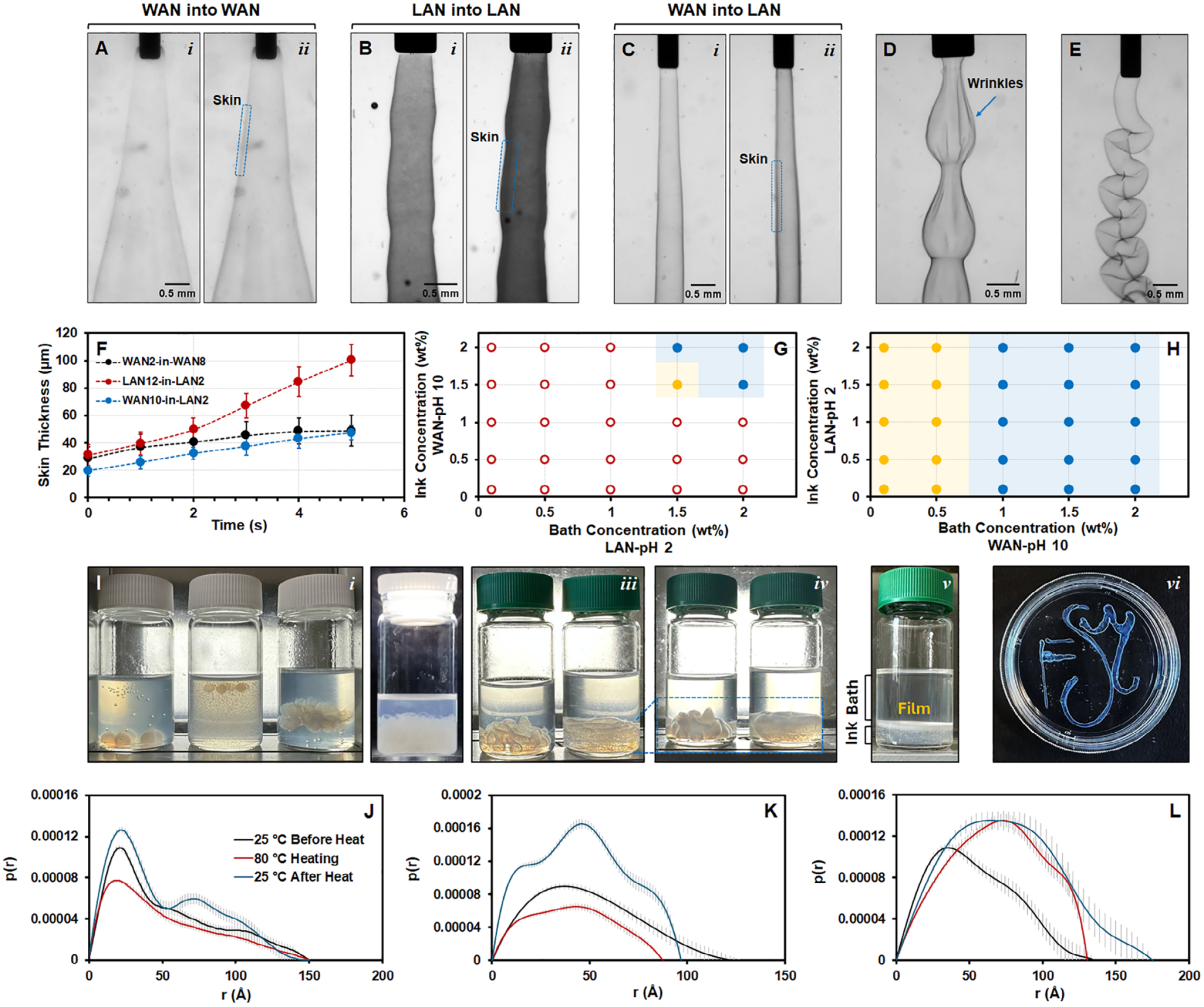
Microscopic view of the non‐equilibrium‐shaped tubular interfacial complex coacervates formed upon extruding (A) WAN (pH 2.0) into WAN (pH 8.0), (B) LAN (pH 12.0) into LAN (pH 2.0), and (C) WAN (pH 10.0) into LAN (pH 2.0), i: initially and ii: after 10 s. (D) formation of wrinkles on tubular interfacial complex coacervates formed via extruding WAN (pH 10.0) into LAN (pH 2.0) with pendant droplet‐shaped sections, indicating the formation of a robust all‐amyloid skin. (E) Formation of flexible tubular interfacial complex coacervates upon controlled extrusion of WAN (pH 10.0) into LAN (pH 2.0). (F) Time‐dependent change in the skin thickness for the analyzed interfacial complex coacervation systems. Concentration‐dependent complexation phase diagram when extruding (G) WAN (pH 10.0) into LAN (pH 2.0) and (H) LAN (pH 2.0) into WAN (pH 10.0) at various inks’ and complexation baths’ concentrations; the phase diagrams divided into three regions including red hollow circles (no capsule formed), yellow circles (capsule formed but difficult to shape), and blue circles (well‐defined capsule formation). (I) i: Formation of interfacial complex coacervate capsules via extruding WAN (pH 2.0) into WAN (pH 8.0) (left vial), LAN (pH 12.0) into LAN (pH 2.0) (middle vial), and WAN (pH 10.0) into LAN (pH 2.0) (right vial); ii: formation of non‐equilibrium‐shaped tubular interfacial complex coacervate upon extruding WAN (pH 10.0) into LAN (pH 2.0); iii: initial formation of capsules (left vial) and tubular (right vial) interfacial complex coacervates upon extruding LAN (pH 2.0) into WAN (pH 10.0), and iv: after heating at 80°C for 10 min; v: formation of a phase‐separated system and a thick interfacially complexed layer at the interface upon extruding LAN (pH 2.0, 2 wt%) into WAN (pH 10.0, 1 wt%), followed by heating at 80°C for 10 min; vi: liquid‐in‐liquid writing upon controlled extrusion of WAN (pH 10.0) into LAN (pH 2.0), FSM stands for the Food and Soft Materials research group. PDDF extracted from SAXS analyses for interfacial complex coacervates formed via extruding (J) WAN (pH 2.0) into WAN (pH 8.0), (K) LAN (pH 12.0) into LAN (pH 2.0), and (L) WAN (pH 10.0) into LAN (pH 2.0), initially upon complexation, after heating at 80°C for 30 min, and cooling down to room temperature; in these figures, all concentrations were set 2 wt% unless otherwise stated.

The wrinkles formed during controlled tubules’ contraction, together with the sectioned pendant‐drop morphology, indicate the formation of a viscoelastic and robust skin that preserves the integrity of the construct under applied forces and suppresses instabilities (Figure 2D and Video S4). The formed tubular structures also exhibited flexible, controllable bending, enabling on‐demand adjustment of their morphology (Figure 2E and Video S5). Microscopic image analysis over time (Figure 2F and Videos S1–S3) showed a progressive increase in the thickness of the amyloid nanofibril complexation region, that is, the skin formed at the liquid–liquid interface. In all systems, complexation occurred immediately after extrusion of the ink into the complexation bath, followed by continuous thickening of the interfacial skin over time. Among the systems studied, the LAN into LAN system showed the fastest increase in skin thickness until equilibrium was reached. This faster thickening may result from the larger charge density difference between oppositely charged lysozyme amyloid nanofibrils (Figure 1I), which provides a stronger electrostatic driving force for interfacial complexation.

Assessing the concentration‐dependent complexation phase diagram for the WAN into LAN complexation system shows an asymmetric trend (Figures 2G,H, S12, and S13). Extrusion of the WAN ink into the LAN complexation bath resulted in the formation of interfacial complex coacervate capsules only when the WAN ink concentration reached 2 wt%, and the LAN bath concentration exceeded 1.5 wt%. Stable capsules did not form outside these concentration ranges. In contrast, extrusion of LAN ink into the WAN complexation bath produced visually stable macroscopic constructs at all concentrations tested. However, reliable control over capsule morphology was achieved only when the WAN bath concentration was above 1 wt%.

The design flexibility of all‐amyloid interfacial complex coacervate systems enables the fabrication of a wide range of macroscopic soft constructs with controllable morphological features. This is supported by a thin skin forming at the liquid–liquid interface upon complexation of oppositely charged amyloid nanofibrils (Figure S14). The formed assemblies involve interfacial complex coacervate capsules (Figure 2Ii) and hollow tubes (Figure 2Iii) using various complexation systems. Interfacial complex coacervate capsules and tubes can also turn into a single monolithic structure after heating at 80°C for 10 min when extruding LAN ink into the WAN complexation bath (Figure 2Iiii, iv). Moreover, extruding LAN ink (2 wt%, pH 2.0) into the WAN complexation bath (1 wt%, pH 10.0), followed by a similar heat treatment, yields a phase‐separated domain with a thick interfacially complexed layer between the phases that thickens over time (Figure 2Iv). Such a wide range of liquid‐in‐liquid structuring, along with the ability to perform liquid‐in‐liquid writing with controllable 3D spatial morphology (Figure 2Ivi), positions all‐amyloid interfacial complex coacervate systems as a versatile approach for on‐demand engineering of macroscopic soft constructs toward advanced functions.

The SAXS Pair Distance Distribution Function (PDDF) analysis for the interfacial complex coacervates created via extruding WAN (pH 2.0) into WAN (pH 8.0) (Figures 2J and S15A) indicates that at 25°C the formed assemblies exhibit dense hierarchically structured protein clusters inside the complexed network. The structure displays a characteristic diameter of approximately 2 nm and a maximum dimension of about 14 nm, corresponding to the rod length. Upon heating to 80°C, the PDDF indicates that the amyloid fibrils become more aligned and ordered within the interfacial complex coacervate, stacking along the longest dimension. This is supported by the appearance of two additional peaks at approximately 5.5 nm and 10.5–11 nm, which likely correspond to distances equivalent to the lateral association of two and three nanofibrils. After cooling back to 25°C, the structure appears to consolidate, with pronounced peaks at approximately 2 and 7.5 nm. The increased intensity of these peaks relative to the 80°C state indicates the formation of a more rigid and stabilized internal structure following the heating–cooling process.

For the LAN (pH 12.0) extruded into LAN (pH 2.0) samples (Figures 2K and S15B), the internal structure differs significantly from that observed for the WAN into WAN system. The PDDF indicates a more globular than elongated organization, which persists even at 80°C. Interestingly, after cooling back to 25°C, three peaks appear in the PDDF profile, suggesting that heating followed by cooling induces the formation of internal rigid domains. The structural analysis shows a dominant peak at approximately 4.5 nm, indicating that this distance is frequently observed within the structure and likely corresponds to the radius of protein clusters within the formed amyloid nanofibril network. Two additional, less intense peaks at approximately 2 and 8 nm are also observed, which may correspond to local contact distances or internal domain spacing and to dimeric/oligomeric associations (∼2 nm × multiple units), respectively.

The WAN (pH 10.0) extruded into the LAN (pH 2.0) system (Figures 2L and S15C) displays an intermediate structural behavior. At 25°C, the internal protein clusters appear relatively elongated, with an approximate diameter of 3 nm and a maximum length of about 14 nm. Upon heating to 80°C, the structure undergoes significant changes, and the clusters adopt a more globular conformation with an estimated radius of around 7 nm. However, the presence of a peak near 11 nm suggests that structural rearrangement is still ongoing at this stage, or the possible presence of oligomers. After cooling back to 25°C, the clusters become fully globular, with a stabilized radius of approximately 6–7 nm. These outcomes demonstrate the ability to alter the hierarchical network at the nanoscale through simple changes in processing parameters or materials.

### 2.2 Functionalization of Amyloid Nanofibrils Interfacial Complex Coacervation

We next developed a simple room‐temperature functionalization method to produce robust and structurally stable all‐amyloid nanofibril interfacial complex coacervates without heating. The process is initiated by functionalizing the WAN with glutaraldehyde (WAN‐Glut) or with a more biocompatible molecule such as genipin (WAN‐Geni) via reaction with the WAN primary amines (FTIR analysis in Figure S16) and employing the LAN in the complexation bath. The functionalization occurred upon controlled addition of various concentrations of glutaraldehyde and genipin (1–15 wt%, relative to amyloid nanofibrils solid content) to WAN dispersion (pH 9.0, 2 wt%), followed by 30 min of stirring for glutaraldehyde, and 1 h stirring plus 72 h incubation in the refrigerator for genipin to complete the functionalization, yielding a blue colored dispersion (Figures S2 and S3).

Glutaraldehyde functionalization proceeds through reaction of WAN primary amines, mainly lysine ε‐amines and N‐terminal groups, with carbonyl groups to form imine linkages via a Schiff‐base reaction (Figure 3Ai). Genipin functionalization proceeds through amine‐initiated ring opening of the dihydropyran structure, followed by condensation and rearrangement reactions that yield a stable heterocyclic linkage (Figure 3Aii). Electrophoretic mobility measurements showed that both WAN‐Glut and WAN‐Geni possess a net negative charge across various crosslinker loadings (1–15 wt%). Increasing the glutaraldehyde concentration enhanced the net negative charge, whereas in the genipin case, the net negative charge remained within a relatively narrow range. This difference could be attributed to the distinct crosslinking mechanisms of glutaraldehyde and genipin with WAN, which differently modify surface‐accessible primary amines, thereby altering the distribution of ionizable functional groups at the nanofibril‐solution interface and influencing the electrokinetic potential at the slipping plane and the measured electrophoretic mobility. In both systems, the reaction pH remained near neutral after functionalization (Figure 3B,C).

**FIGURE 3:**
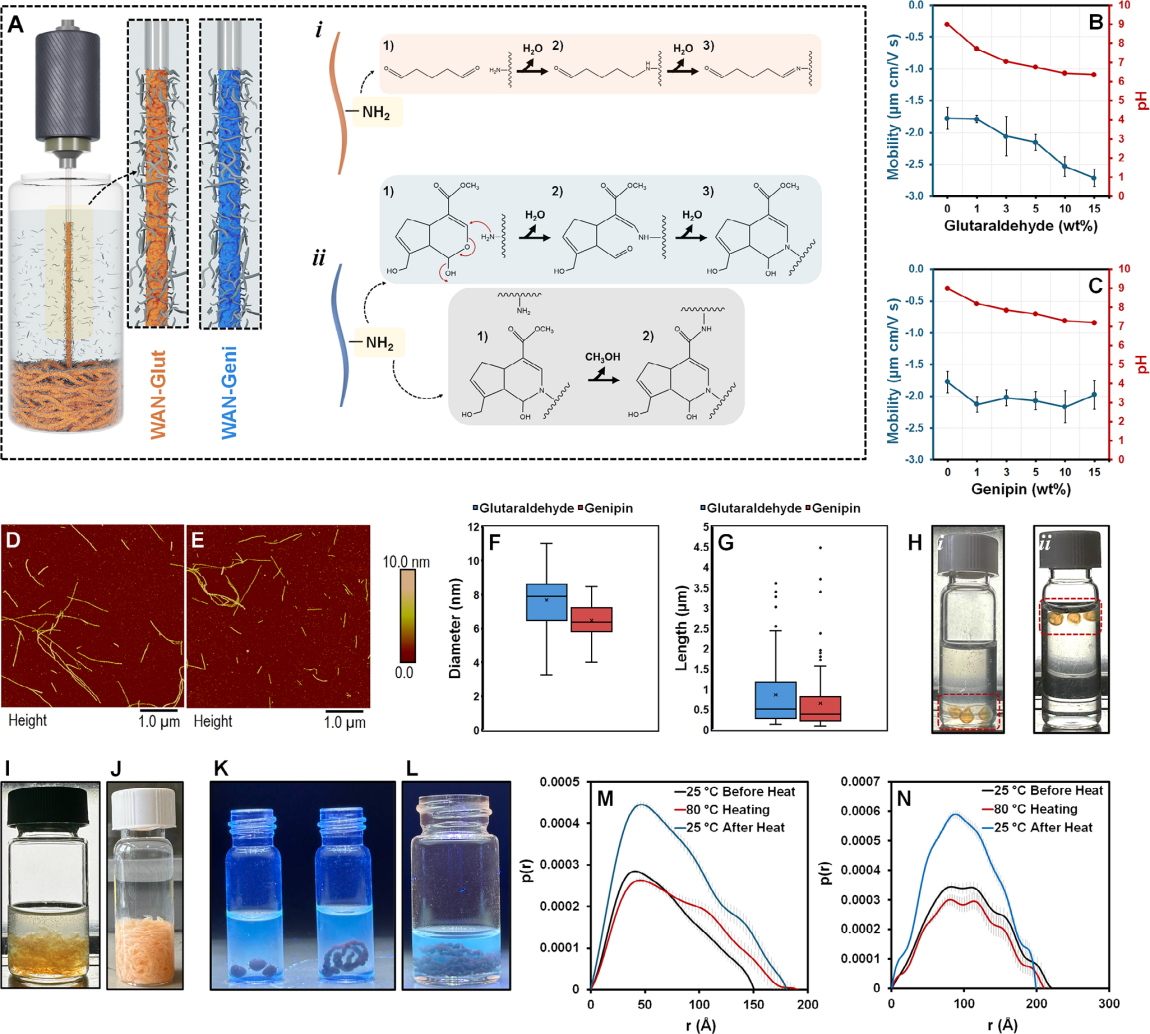
(A) Schematic illustration of the WAN functionalization via i: glutaraldehyde and ii: genipin, leading to more robust and stable proteinaceous interfacial complex coacervates. Electrophoretic mobility and pH analysis of functionalized WAN with various concentrations of (B) glutaraldehyde and (C) genipin. AFM analysis of (D) glutaraldehyde‐ and (E) genipin‐functionalized WAN. (F) Diameter and (G) length distribution of functionalized WAN with glutaraldehyde and genipin. (H) i: Formation of interfacial complex coacervate capsules upon extruding WAN‐Glut ink into the LAN complexation bath (pH 2.0, 2 wt% plus 20 vol% isopropyl alcohol), followed by ii: short aging and transfer into water. (I) Forming non‐equilibrium‐shaped interfacial complex coacervate tubes upon extruding WAN‐Glut ink (pH 10.0, 2 wt%) into the LAN complexation bath, and (J) when transferred to water after short aging. (K) Formation of interfacial complex coacervate capsule/tube and (L) non‐equilibrium‐shaped tubes upon extruding WAN‐Geni ink (pH 10.0, 2 wt%) into the LAN complexation bath, emitting red color under exposure to UV. SAXS PDDF analyses for formed assemblies via extruding (M) WAN‐Glut and (N) WAN‐Geni into the LAN complexation bath, at various temperatures.

AFM analysis showed that both WAN‐Glut and WAN‐Geni retained their nanofibrillar morphology after functionalization, while exhibiting a considerable decrease in contour filament length and a concurrent increase in fibril diameter relative to untreated WAN due to functionalization (Figure 3D–G). WAN‐Glut exhibited a mean diameter of 7.69 ± 1.58 nm (range: 3.25–11.00 nm) and a mean length of 0.86 ± 0.65 µm (range: 0.13–3.61 µm). In comparison, WAN‐Geni showed a diameter distribution of 6.47 ± 1.04 nm (range: 3.99–8.47 nm) and a length distribution of 0.80 ± 0.75 µm (range: 0.09–4.48 µm). The functionalization process also decreased the nanofibril aspect ratio by approximately one order of magnitude relative to untreated WAN, yielding *L*_c_/*D* values of ∼1.12 × 10^2^ and ∼1.24 × 10^2^ for WAN‐Glut and WAN‐Geni, respectively. The increased diameter and reduced contour length observed after functionalization likely result from crosslinker‐mediated lateral association or partial fusion of amyloid nanofibrils. These effects, together with fragmentation during the reaction and handling steps, result in a marked decrease in the aspect ratio.

Functionalized amyloid nanofibrils enabled the formation of robust, interfacial complex coacervates with controllable morphologies and optimal stability, without the need for post‐heating after complexation. For instance, extruding WAN‐Glut ink (pH 10.0, 2 wt%) into a LAN complexation bath (pH 2.0, 2 wt% with 20 vol% isopropyl alcohol) produced capsules (Figure 3Hi, ii) and non‐equilibrium‐shaped interfacial complex coacervate tubes (Figure 3I). After short aging, these structures could be transferred into water (Figure 3Hii,J) or water‐washed for several cycles to remove any excess crosslinker. This highlights the stability and practicality of WAN‐Glut‐based interfacial complex coacervates for advanced functional applications. Similar constructs were formed upon extruding WAN‐Geni ink (pH 10.0, 2 wt%) into the same complexation bath, emitting red color upon UV exposure owing to the presence of genipin molecules in the structure (Figure 3K,L).

The SAXS analysis shows that the protein cluster structure formed when WAN‐Glut (pH 10.0, 2 wt%) is extruded into the LAN complexation bath (Figures 3M and S17A) adopts a relatively elongated shape at 25°C, with a short‐axis diameter of approximately 3–4 nm and a maximum dimension of about 15 nm. Heating to 80°C promotes the stacking of protein clusters, favoring their alignment along the long axis of the rod‐like structures. This behavior is reflected in peaks at approximately 10 and 15 nm, which likely correspond to the association of two and three rods, respectively. The increase in peak intensity observed after the heating and cooling back to 25°C suggests that this thermal treatment enhances the organization and packing of protein clusters within the formed network.

In contrast, when WAN‐Geni (pH 10.0, 2 wt%) is extruded into the LAN complexation bath (Figures 3N and S17B), the SAXS PDDF at 25°C indicates the presence of two populations of globular protein clusters with characteristic radii of approximately ∼7 and ∼12 nm, both occurring in similar proportions. The broader peaks observed at around ∼15 and ∼20 nm may correspond to oligomeric assemblies of these structures. Heating to 80°C increases the rigidity and organization of the structure, revealing the presence of smaller internal domains at approximately 1 and 4 nm. After cooling back to 25°C, one population of protein clusters becomes more dominant, with a characteristic radius of approximately 8–9 nm.

Fluorescent microscopy revealed the pH‐dependent stability of the functionalization‐assisted amyloid nanofibril interfacial complex coacervation, highlighting its ability to form and dissociate in response to external stimuli. Upon extruding WAN‐Geni (pH ∼7.3, 2 wt%) prior to pH adjustment into the LAN complexation bath (pH 2.0, 2 wt% plus 20 vol% isopropyl alcohol), a tubular interfacial complex coacervate formed instantly. However, the tubular construct lost its stability after 2 min, leading to dissociation of the formed assemblies into the complexation bath (Figure 4A). Whereas post‐synthesis pH adjustment to 10.0 maintained the stability of the tubular interfacial complex coacervate (Figure 4B) with a well‐defined all‐amyloid nanofibril assembly at the liquid–liquid interface (Figure S18). This highlights the necessity of pH adjustment to achieve stable interfacial complex coacervates. A similar trend was observed when forming a tubular interfacial complex coacervate by extruding WAN‐Glut (pH 10.0, 2 wt%) in the same complexation bath (pH 2.0, 2 wt% plus 20 vol% isopropyl alcohol), yielding stable tubular constructs over time via pH adjustment (Figure 4C).

**FIGURE 4:**
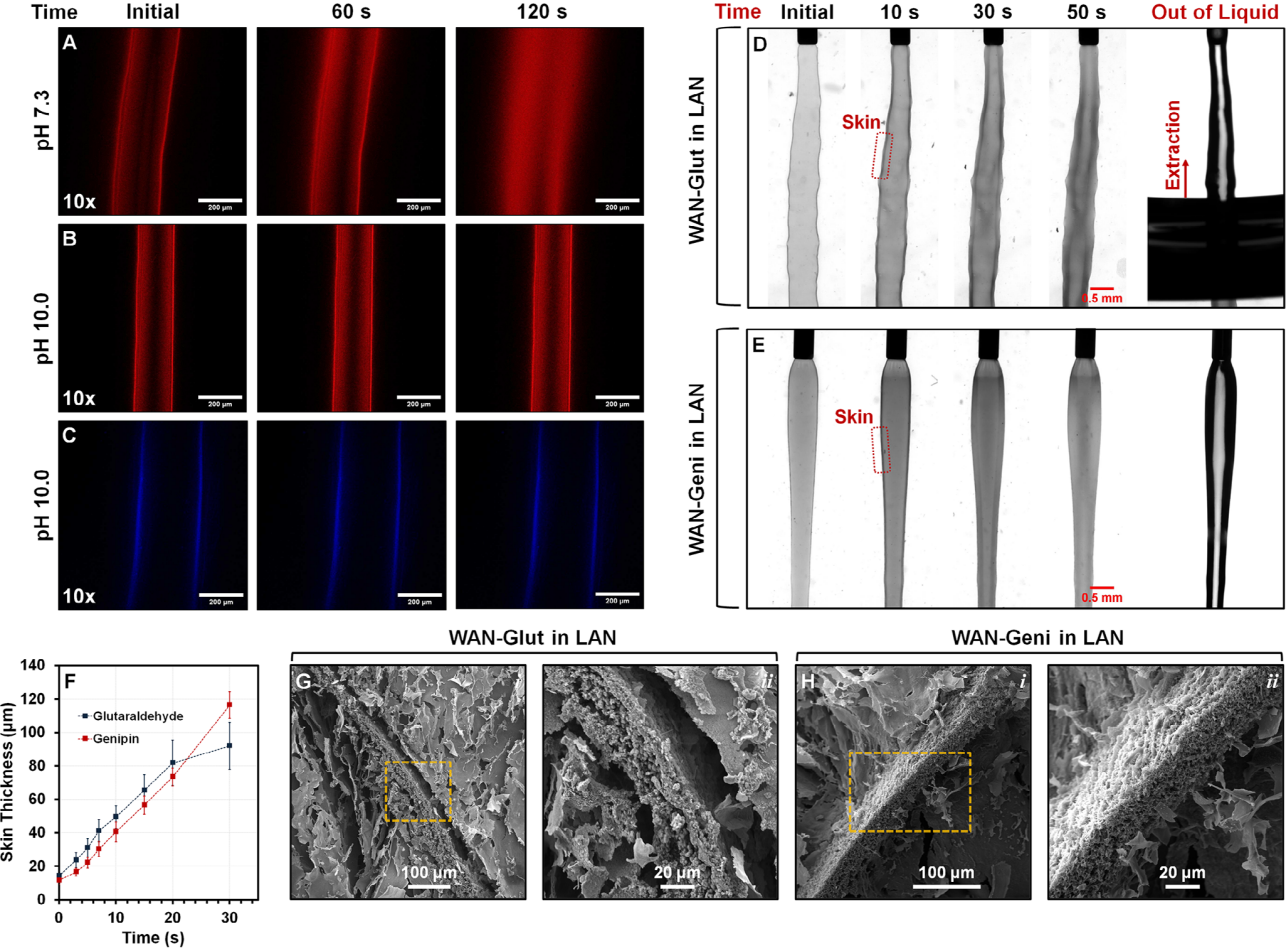
Fluorescent microscopy images of tubular interfacial complex coacervate formed through the complexation of WAN‐Geni at (A) pH 7.3 and (B) pH 10.0 with LAN at pH 2.0 at different times. (C) Fluorescent microscopy images of tubular interfacial complex coacervate formed via the complexation of WAN‐Glut (pH 10.0, 2 wt%) with LAN in the complexation bath (pH 2.0, 2 wt% plus 20 vol% isopropyl alcohol) at different times; the excitation wavelength was set at 405 nm. Formation of protein tubes akin to an artificial vein when extruding (D) WAN‐Glut (pH 10.0, 2 wt%) and (E) WAN‐Geni (pH 10.0, 2 wt%) into the LAN complexation bath. (F) Change in the skin thickness of the formed protein tubes over time. FESEM images of the skin formed upon complexation of (G) i–iii: WAN‐Glut and (H) i–iii: WAN‐Geni with LAN at different magnifications, after freezing and lyophilization of assemblies; part ii is a magnified view of the marked area in part i.

The observed pH‐dependent stability could arise from electrostatic interactions between oppositely charged amyloid nanofibrils, or from a re‐equilibration of pH at pHs intermediate between the extruding and bath solutions. At lower pH, the reduced stability may be caused by proton diffusion from the acidic bath into the interfacial complex coacervate tube. This could shift the local environment toward charge neutralization, weaken electrostatic interactions, and promote redissolution of the amyloid nanofibrils into the surrounding medium. Readjusting the pH to 10.0 likely increases and stabilizes the net negative charge of the functionalized WAN, therefore stabilizing the formed assemblies.

The complexation of functionalized WAN with LAN at the liquid–liquid interface enables the formation of all proteinaceous tubular compartments, analogous to veins or artificial vascular systems. Extruding WAN‐Glut (pH 10.0, 2 wt%) (Figure 4D and Video S6) or WAN‐Geni (pH 10.0, 2 wt%) (Figure 4E and Video S7) into the LAN complexation bath enables the formation of tubular proteinaceous compartments with tailored 3D spatial morphology. Depending on the availability of the complexing species in the medium and their diffusion length scale, the thickness of the complexed network continues to grow (Figure 4F), enabling time‐dependent control of skin thickness.

The FESEM analyses revealed the formation of a hierarchically structured, interconnected nanofibrillar network via complexation of oppositely charged functionalized amyloid nanofibrils (Figure 4G,H). In both WAN‐Glut (Figures 4G, S19, and S20) and WAN‐Geni (Figures 4H and S21) systems, this interfacial network acted as a barrier between the aqueous phases. It stabilized the interfacial complex coacervates, supported LLPS, and preserved the morphology of the extruded ink. As a result, the system allowed precise morphological control and the formation of all‐amyloid proteinaceous interfacial complex coacervates with programmable architectures and tailored 3D organization.

### 2.3 Soft Bioelectronic and Shielding Proteinaceous Interfacial Complex Coacervates

The all‐amyloid interfacial complex coacervation strategy, based on charge‐mediated and/or functionalization‐assisted complexation of oppositely charged amyloid nanofibrils, provides control over composition, morphology, and material properties across multiple length scales. This design flexibility expands the scope of proteinaceous compartments and supports the development of amyloid‐based materials for emerging device applications.

These materials can further be made electrically conductive by incorporating multi‐walled carbon nanotubes (MWCNTs) and/or poly(3,4‐ethylenedioxythiophene):poly(styrene sulfonate) (PEDOT:PSS) within them. Ionic conductivity can also be increased by adding salt (e.g., KCl). In contrast to conventional complex coacervate systems, where salt‐induced screening weakens long‐range electrostatic interactions and destabilizes the assemblies, the functionalized amyloid nanofibrils are stabilized by multiple complementary interaction pathways, including electrostatic, hydrogen‐bonding, hydrophobic, and covalent bonding, thereby reducing their dependence on electrostatic interactions alone for structural integrity. These features enable robust all‐amyloid interfacial complex coacervates for soft electronics, iontronics, and electromagnetic (EM) shielding applications. Adding non‐functionalized MWCNTs (0.5–2 wt%) to a WAN dispersion (pH 10.0, 2 wt%) produced uniform and stable aqueous colloidal dispersions (Figure 5A) [47]. This avoided the need for costly and time‐consuming carbon nanotube functionalization or purification to achieve water dispersibility (FTIR analysis, Figure S22). Air‐drying the WAN‐MWCNT dispersions on glass substrates formed a bicontinuous arrangement whose structure can be further tuned by varying the MWCNT concentration (Figures 5B and S23).

**FIGURE 5:**
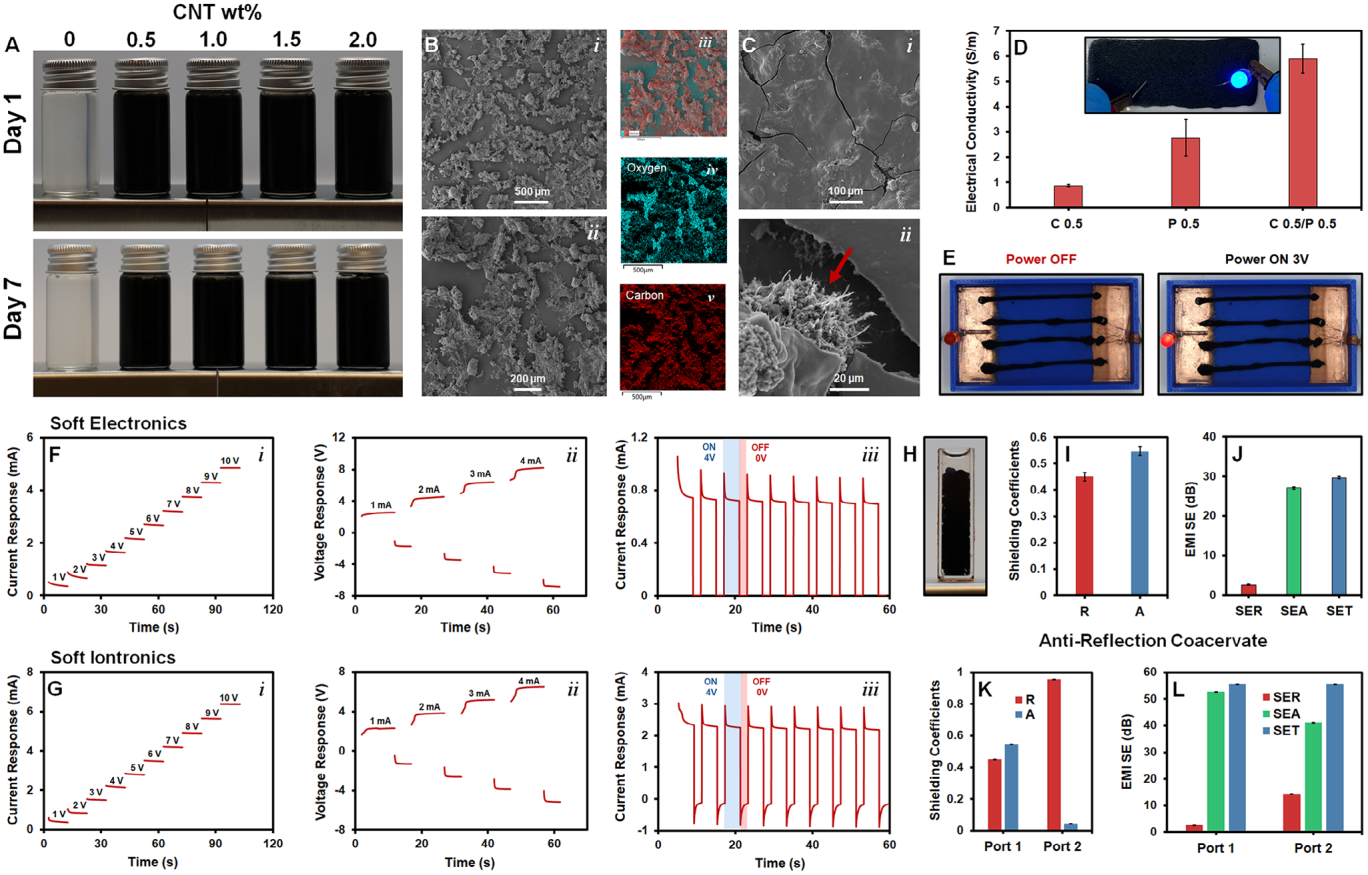
(A) Colloidal stability of various concentrations (0.5–2 wt%) of non‐functionalized MWCNT dispersions in WAN (pH 10.0, 2 wt%), initially and after 1 week. (B) i and ii: FESEM images of WAN‐MWCNT bicontinuous arrangements formed via drying the reinforced sample with 0.5 wt% MWCNT on a glass substrate; parts iii–v show the EDS analysis and respective elemental distribution maps. In parts iii–v, the red and blue colors represent carbon and oxygen, respectively. (C) i and ii: FESEM images of the conductive film made from WAN‐MWCNT:PEDOT:PSS ink. (D) Electrical conductivity of the bicontinuous WAN‐MWCNT arrangement (C 0.5), WAN‐PEDOT:PSS film (P 0.5), and its hybrid form WAN‐MWCNT:PEDOT:PSS (C 0.5/P 0.5); the inset shows the ability of WAN‐MWCNT:PEDOT:PSS thin film to light up an LED at an applied voltage of about 3 V. (E) Electrically conductive liquid wires coacervates formed via extruding WAN‐MWCNT:PEDOT:PSS ink into the LAN complexation bath and its ability to light up an LED at an applied voltage of 3 V. (F) Soft electronics responses of formed electrically conductive liquid wires, including i: its current response at different applied voltages (1–10 V), ii: its voltage response at various applied currents (1–4 mA), and iii: cyclic on‐off response at 4–0 V. (G) Soft iontronic responses of ionically conductive liquid wires formed via extruding reinforced WAN‐Glut with 0.5 M KCl into the LAN complexation bath, including i: its current response at different applied voltages (1–10 V), ii: its voltage response at various applied currents (1–4 mA), and iii: cyclic on‐off response at 4–0 V. (H) Forming electrically conductive tubular interfacial complex coacervates in a quartz cuvette with an internal width of 10 mm via extruding the WAN‐MWCNT:PEDOT:PSS ink in the LAN complexation bath, and its electromagnetic shielding character, including (I) shielding coefficients (reflectance (R) and absorbance (A)) and (J) EMI SE parameters (reflection loss (SE_R_), absorption loss (SE_A_), total shielding effectiveness (SE_T_)). (K) Shielding coefficient and (L) EMI SE when one side of the cuvette is covered with a ∼0.39 mm‐thick Ti mesh, highlighting the anti‐reflecting performance of the as‐developed conductive interfacial complex coacervates.

While the neat WAN thin film (2 wt%) exhibits a hydrophilic nature, with a contact angle of 85.30 ± 0.73°, increasing the MWCNT concentration (from 0.5 wt% to 2 wt%) shifts the nature of the construct toward greater hydrophobicity, with a contact angle of 102.80 ± 3.02° at 0.5 wt% MWCNT. This further increases to a maximum of 125.72 ± 3.47° at an MWCNT loading of 1.5 wt% (Figure S24). Dynamic contact angle measurements reveal rapid liquid uptake, where the MWCNT‐containing films initially present contact angles above 100°, followed by a gradual decline as water penetrates the network (Figure S24 and Video S8). This amphiphilic response arises from the combination of hydrophobic MWCNT domains and the hydrophilic amyloid‐nanofibrillar matrix, together with hierarchical roughness introduced by the bicontinuous MWCNT network.

At lower MWCNT concentrations (0.5 wt%), the carbonaceous blend exhibited a well‐connected network with narrower microscale channels, as evidenced by FESEM and EDS analyses (Figures 5B and S25A). However, further increasing the MWCNT loading beyond 1 wt% led to the formation of larger MWCNT aggregates and wider bicontinuous channels (Figures S23 and S25B–D). This results in a concentration‐dependent decrease in electrical conductivity due to reduced network connectivity. This was confirmed by a sharp decline in electrical conductivity from 0.87 ± 0.04 S/m to 0.005 ± 0.001 S/m when the MWCNT concentration changed from 0.5 wt% to 1 wt%. Adding 0.5 wt% PEDOT:PSS along with 0.5 wt% MWCNT to the amyloid nanofibril dispersion facilitated the CNT‐to‐CNT contact (Figures 5C, S26, and S27). This occurs because PEDOT:PSS interacts with both amyloid nanofibrils and MWCNTs, and the resulting interconnection could be due to π–π interactions with MWCNTs and electrostatic or hydrogen‐bonding interactions with the nanofibrils. This results in a more uniform conductive thin film capable of lighting an LED at an applied voltage of ∼3 V with an improved electrical conductivity of 5.90 ± 0.57 S/m when hybridized (Figure 5D).

While the dried WAN dispersion (pH 10.0, 2 wt%) on a glass substrate forms a uniform and interconnected nanofibrillar film (Figure S28), drying the WAN dispersion after adding 0.5 wt% PEDOT:PSS leads to a partially disconnected network (Figures S29 and S30), showing the superiority of hybridization with MWCNT to achieve a more interconnected network. Using the WAN‐MWCNT (0.5 wt%) (Figures S31 and S32) ink or WAN‐MWCNT:PEDOT:PSS (0.5:0.5 wt%) (Figures S33 and S34) hybrid conductive ink enables the formation of a proteinaceous conductive interfacial complex coacervate thin film upon contact with the LAN complexation bath, with visible surface wrinkles upon complexation (Figure S33).

The design's flexibility enables the incorporation of amyloid nanofibril‐based inks with conductive or ionic additives to develop soft electronic or iontronic systems. To this end, a parallel soft electronic circuit was developed by connecting two copper electrodes with four electrically conductive tubular interfacial complex coacervates formed by extruding WAN‐MWCNT:PEDOT:PSS conductive ink into the LAN complexation bath, capable of lighting an LED at an applied voltage of 3 V (Figure 5E). The as‐developed soft circuit showed a controllable current response as the voltage was increased from 1 V to 10 V in 1 V steps, with current values ranging from 0.35 mA to 4.85 mA (Figure 5Fi). Additionally, the response potential could be tuned to anodic (positive) or cathodic (negative) values by changing the magnitude and direction of the applied current, enabling reversible electrical polarization. This is essential for adaptive electronics and functional materials that require dynamic switching between oxidation and reduction states. Accordingly, increasing the applied current from 1 mA to 4 mA, with the current direction set to positive and negative, yielded potential responses of 2.59–8.20 V and −1.75 to −6.87 V, respectively (Figure 5Fii). The circuit also exhibited a stable on/off cycling response when the voltage was increased to 4 V and then decreased to 0 V (Figure 5Fiii).

Similar behavior was also observed in an iontronic soft circuit, in which charge transport is carried by ions. The circuit was fabricated by extruding four non‐equilibrium‐shaped tubular interfacial complex coacervates between two copper electrodes using WAN‐Glut ink (pH 10.0, 2 wt%) containing 0.5 M KCl, which can light an LED at an applied voltage of 3 V (Figure S35). The WAN‐Glut ink exhibited an ionic conductivity of 6.44 ± 0.11 mS/cm, which was improved to 59.30 ± 0.38 mS/cm upon adding 0.5 M KCl without screening the amyloid complexation upon controlled extrusion into the complexation bath. The soft iontronic circuit exhibited controllable current responses ranging from 0.37 mA to 6.36 mA as the voltage was increased from 1 V to 10 V in 1 V steps (Figure 5Gi). It also showed reversible polarization and controllable potential responses at applied currents ranging from 1 mA to 4 mA, with potential responses of 2.31–6.50 V and −1.31 to −5.16 V at positive and negative current directions (Figure 5Gii).

Such controllable responses are also accompanied by stable current and potential responses over time when applying a 3 V voltage or a 1 mA current to the iontronic soft circuit (Figure S36). The cyclic on/off response of the soft iontronic circuit, obtained by switching the voltage between 4 V and 0 V, differs markedly from that of an electronically conductive circuit. During voltage switching, mobile ions migrate and accumulate at the electrode interfaces, forming electric double layers and space‐charge regions within the material. When the voltage is set to 0 V, this stored ionic charge drives a transient reverse potential, producing a negative signal that gradually returns to zero as the ions redistribute and the system relaxes (Figure 5Giii).

The electrically or ionically conductive amyloid interfacial complex coacervates also showed promising potential as effective absorption‐dominant EM shields. Extruding electrically conductive WAN‐MWCNT:PEDOT:PSS ink into the LAN complexation bath formed a tubular interfacial complex coacervate that serves as a potent EM shield and anti‐reflector (Figure 5H). The resulting construct showcased effective EM dissipation with an absorption‐dominant character (absorbance (*A*): 0.54) and a total EM shielding effectiveness (SE_T_) of 29.71 dB, blocking ∼99.9% of the incident EM waves power (Figure 5I,J). Notably, when *A* > 0.5 or *A* < 0.5, the shielding system is defined as absorption‐dominant or reflection‐dominant, respectively. When placing the conductive proteinaceous interfacial complex coacervate formed within a cuvette, paired with a conductive Ti mesh (∼0.39 mm thick) between a two‐port waveguide adapter, with the proteinaceous interfacial complex coacervate facing port one and the Ti mesh facing port two, the paired system exhibits asymmetric EM shielding. The paired shielding system exhibits an SE_T_ of 55.49 dB (blocking ∼99.9997% of incident EM waves power), with absorption‐dominant (*A*: 0.54) and reflection‐dominant (*A*: 0.04) characteristics at ports one and two, respectively (Figure 5K,L). These results indicate that proteinaceous interfacial complex coacervates can serve as effective, low‐cost EM‐shielding and anti‐reflective materials.

The ionically conductive soft amyloid nanofibril interfacial complex coacervates also showed potential as absorption‐dominant shielding systems. Extruding ionic WAN‐Glut ink (pH 10.0, 2 wt%, 0.5 M KCl) into the LAN complexation bath and aging for 1 day yields a tubular ionic interfacial complex coacervate (Figure S37). When placed in an 8 mm‐thick mold (diameter 3 cm) and positioned between waveguide adaptors, the coacervate acted as an absorption‐dominant shield, with an absorbance (A) of 0.63 and an SE_T_ of 42.14 dB, capable of blocking ∼99.9939% of the incident EM wave power.

### 2.4 Viscoelastically Tunable Glow‐in‐the‐Dark Amyloid Nanofibril Interfacial Complex Coacervates

The engineerable nature of the all‐amyloid interfacial complex coacervates enabled easy tuning of their viscoelastic properties, while maintaining macromolecular crowding via stable, uniform microparticle suspensions or by adding high solid content to the interfacial complex coacervate. As a proof of concept, mild (3‐aminopropyl)trimethoxysilane (APTMS) treatment of SrAl_2_O_4_:Eu^2+^, Dy^3+^ (APTMS‐SrAl_2_O_4_:Eu^2+^, Dy^3+^; see Experimental Section), a persistent luminescent pigment with long afterglow and an average particle size of ∼50 µm (according to the supplier), enabled its uniform distribution within the WAN ink (pH 10.0, 2 wt%). This was achieved through viscoelastic enhancement of the ink via reinforcement with varying concentrations of hydroxypropyl methylcellulose (HPMC, 1–5 wt%) prior to the addition of APTMS‐SrAl_2_O_4_:Eu^2+^, Dy^3+^ to the ink. Extruding the modified ink into the LAN complexation bath enables the formation of a glow‐in‐the‐dark amyloid nanofibril interfacial complex coacervate with controllable morphological features (Figure 6Ai, ii).

**FIGURE 6:**
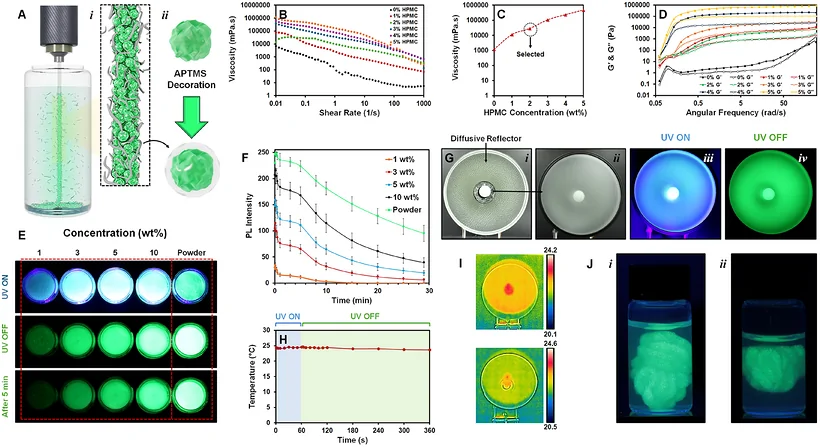
(A) Formation of viscoelastically tunable glow‐in‐the‐dark amyloid nanofibril tubular interfacial complex coacervate upon extruding APTMS‐SrAl_2_O_4_:Eu^2+^, Dy^3+^ containing WAN (WAN: pH 10.0, 2 wt%; HPMC: 2 wt%) into the LAN complexation bath capable of i: stabilizing large APTMS‐SrAl_2_O_4_:Eu^2+^, Dy^3+^ microparticles (size ∼50 µm) via ii: complexation of APTMS‐SrAl_2_O_4_:Eu^2+^, Dy^3+^ with negatively charged WAN within the tubular construct and WAN with LAN at the liquid–liquid interface. (B) Flow curve of the WAN (pH 10.0, 2 wt%) inks upon reinforcement with various HPMC concentrations (1–5 wt%), (C) related viscosities at a shear rate of 0.1 s^−1^, and (D) frequency sweep test. (E) Glow‐in‐the‐dark capability of the WAN ink (WAN: pH 10.0, 2 wt%; HPMC: 2 wt%) containing various concentrations of APTMS‐SrAl_2_O_4_:Eu^2+^, Dy^3+^ (1–10 wt%) upon UV exposure for 30 s, turning the UV off, and after 30 min; (F) the related time‐dependent PL intensity variation of the glow‐in‐the‐dark amyloid nanofibril ink at various pigment concentrations. (G) Formation of a persistent protein luminescent lamp by placing APTMS‐SrAl_2_O_4_:Eu^2+^, Dy^3+^ enriched amyloid ink in i and ii: a diffusive reflector, followed by iii: UV irradiation, and iv: glow in the dark upon turning the UV off. (H) Change in the temperature of the glow‐in‐the‐dark protein luminescent lamp upon exposure to UV and after turning the UV off, plus (I) the related temperature gradient map upon UV irradiation for 60 s and after 5 min. (J) i: Formation of glow‐in‐the‐dark amyloid interfacial complex coacervate and ii) the ability to extrude and hold in a specific position in the complexation bath.

According to the flow curves (Figure 6B), the hybrid WAN‐HPMC gels exhibited shear‐thinning behavior upon disruption of the gel network under applied shear force. A gradual increase in HPMC concentration from 1 wt% to 5 wt% progressively increased the viscosity from 1.14 × 10^4^ to 4.75 × 10^5^ mPa s at a shear rate of 0.1 s^−1^ (Figure 6C). Frequency sweep analysis (Figure 6D) showed that all HPMC‐reinforced samples displayed gel‐like behavior with storage modulus (*G*′) exceeding loss modulus (*G*″) and a frequency‐independent response beyond 0.5 rad s^−1^; whereas the pristine WAN ink (pH 10.0, 2 wt%) exhibited a frequency‐dependent rheological response. This confirms enhanced gel robustness with increasing HPMC concentration. Strain‐amplitude oscillatory measurements (Figure S38) revealed a progressive increase in *G*′ with increasing HPMC loading until reaching the yield point (*G*′ = *G*″), where the yield stress and corresponding strain amplitude shifted from 33.5 Pa and 21.7% to 1414.2 Pa and 35.0% as HPMC increased from 1 wt% to 5 wt%, respectively. This shift of the solid‐to‐liquid transition to higher moduli and strain amplitudes indicates that the viscoelastic network strengthens with increasing HPMC loading. Similarly, elastic recovery analysis (Figure S39) demonstrated the formation of a resilient gel structure, evidenced by rapid G′ restoration to its initial plateau after each deformation cycle, with recovery improving as HPMC concentration increased.

This allowed uniform suspension of high loadings of APTMS‐SrAl_2_O_4_:Eu^2+^, Dy^3+^ luminescent microparticles (1–10 wt%) in WAN ink by on‐demand alteration of viscoelastic character, yielding glow‐in‐the‐dark protein dispersions that, upon brief exposure to UV light for 30 s, emit green light in the dark for much longer than the exposure time (Figure 6E,F and Video S9). Placing this luminescent protein gel at the center of a diffusive reflector enables the development of a persistent protein luminescent lamp (Figure 6Gi–iv and Video S10) that uniformly reflects emitted green light with negligible temperature increment upon UV exposure (Figure 6H,I). Controlled extrusion of the luminescent proteinaceous ink (WAN: pH 10.0, 2 wt%; HPMC: 2 wt%; APTMS‐SrAl_2_O_4_:Eu^2+^, Dy^3+^: 1 wt%) into the LAN complexation bath allows making a non‐equilibrium‐shaped tubular glow‐in‐the‐dark interfacial complex coacervate, with the ability to extrude and shape in a specific position of the complexation bath (Figure 6Ji, ii and Video S11).

## 3 Conclusions and Outlook

This study introduces an all‐amyloid interfacial complex coacervate strategy that harnesses liquid–liquid phase separation to create functional proteinaceous compartments. The interfacial complex coacervates form through the complexation of oppositely charged amyloid nanofibrils derived from food‐based proteins such as β‐lactoglobulin and lysozyme. The complexation arises from identical or distinct amyloid nanofibril types via charge‐mediated and/or functionalization‐assisted interactions with glutaraldehyde or genipin as crosslinkers. This enables the formation of structurally robust proteinaceous compartments through either post‐complexation thermal stabilization or covalent crosslinking of the assemblies after short aging. This strategy enables the engineering of proteinaceous compartments from the nanometer‐scale building blocks to macroscopic arrangements, allowing the creation of compositionally versatile, morphologically tunable, and structurally robust proteinaceous constructs. Such design flexibility enables the incorporation of functionalities not readily accessible with conventional proteinaceous coacervates, as exemplified by artificial all‐proteinaceous veins/tubules, bioelectronics, iontronics, and bio‐based optics. Beyond this study, the broad engineerability of all‐amyloid interfacial complex coacervation extends the capabilities of protein‐based soft matter, providing a versatile framework for the development of advanced biofabrication platforms, engineered tissue constructs, liquid‐in‐liquid printing, artificial biomolecular condensates, soft bioelectronics, bio‐optical systems, and emerging biomimetic materials and technologies.

## 4 Experimental Section

### 4.1 Materials and Methods

#### 4.1.1 Synthesis of Amyloid Nanofibrils

Amyloid nanofibrils were synthesized using β‐lactoglobulin (isolated whey protein, NZMP, New Zealand) and lysozyme (hen egg white lysozyme, Roche; REF: 10837059001; GTIN: 04038377005393; LOT: 81067121) following well‐established procedures [24, 43]. For the synthesis of whey amyloid nanofibrils (WAN), 2 wt% protein powder was dispersed in ultrapure deionized water (18.2 MΩ cm), and the pH was adjusted to 2.0 with 2 M HCl (37% HCl, Sigma‐Aldrich). The dispersion was stirred at 350 rpm and 90°C for 5 h to facilitate fibrillation, then quenched in an ice bath. A similar process was used to synthesize lysozyme amyloid nanofibrils (LAN), except that the heat‐stir time was 24 h at 350 rpm and 90°C, followed by quenching in an ice bath. The successful synthesis of WAN and LAN was confirmed by birefringence under crossed‐polarized light (Figure S1) and by atomic force microscopy (AFM) analyses.

#### 4.1.2 Functionalization of Amyloid Nanofibrils

The functionalization of the WAN with glutaraldehyde (25% glutaraldehyde solution, Sigma‐Aldrich) was carried out by first adjusting the pH of a 2 wt% WAN dispersion to 9.0 using 0.1 M NaOH solution, followed by dropwise addition of 5 wt% glutaraldehyde aqueous mixture to WAN at concentrations of 1–15 wt%, considering the WAN solid content. The mixture was stirred for 30 min at 350 rpm and at room temperature (Figure S2). The sample was used fresh for further analyses.

The functionalization of the WAN with genipin (Thermo Scientific; CAT: 466640010; LOT: A0469004; CAS:6902‐77‐8) was carried out by first adjusting the pH of a 2 wt% WAN dispersion to 9.0 using 0.1 M NaOH solution, followed by dropwise addition of a 5 wt% genipin aqueous mixture to WAN at concentrations of 1–15 wt%, considering the WAN solid content. The genipin dispersion was prepared by first adding the powder to absolute ethanol (anhydrous ethanol with ≤ 0.003% water; Sigma‐Aldrich) and its uniform dispersion, followed by water addition, as genipin's solubility and stability in water are limited (20 vol% ethanol, 80 vol% water, 5 wt% solid content). The mixture was stirred for 1 h at 350 rpm and room temperature, then incubated for 72 h at 4°C to complete the crosslinking. Upon completion of the functionalization process, a dark blue dispersion was obtained for all samples (Figure S3).

#### 4.1.3 Interfacial Complex Coacervates Optimization and Preparation

All‐amyloid interfacial complex coacervates were formed as capsules or as non‐equilibrium‐shaped tubes through controlled extrusion of either positively or negatively charged amyloid nanofibril dispersion into the oppositely charged amyloid nanofibril complexation bath. The pH of the WAN and LAN, with a fixed concentration of 2 wt%, was varied from 2.0 to 12.0 to assess the effect of pH on the formation of interfacial complex coacervate. Three systems were assessed by extruding WAN into WAN, LAN into LAN, and WAN into LAN. In all cases, the ink was completely aqueous (100 vol% water), and the complexation bath was mixed with 20 vol% isopropyl alcohol prior to extrusion. After complexation, all samples were heated at 80°C for 10 min to improve stability. The concentration‐dependent all‐amyloid interfacial complex coacervates were examined in the WAN into LAN and LAN into WAN systems by fixing the WAN and LAN pH at 10.0 and 2.0, respectively, at concentrations ranging from 0.1 wt% to 2 wt%.

The formation of interfacial complex coacervates with functionalized WAN was carried out by adjusting the pH of the functionalized WAN with glutaraldehyde (WAN‐Glut) or genipin (WAN‐Geni) to 10.0 (WAN concentration 2 wt%, 10 wt% crosslinker, considering WAN solid content), followed by extrusion into the LAN complexation bath (pH 2.0, 2 wt%, 20 vol% isopropyl alcohol).

#### 4.1.4 Soft Electrically or Ionically Conductive Interfacial Complex Coacervates

The electrically conductive amyloid nanofibril ink was prepared by dispersing various concentrations of MWCNT (0.5–2 wt%, NC7000 supplied by Nanocyl SA) into the WAN (pH 10.0, 2 wt%) via stirring for 30 min at 350 rpm, followed by 10 min bath sonication, and another 30 min stirring at 350 rpm. The reinforced WAN with PEDOT:PSS was prepared by adding 0.5 wt% freeze‐dried PEDOT:PSS (Heraeus; Clevios PH1000) (24 h freezing at −80°C, 48 h freeze‐drying using a benchtop LABCONCO freeze‐drier) and 5 vol% DMSO (Sigma‐Aldrich) to the WAN dispersion, followed by the same dispersing protocol. The hybrid reinforcement of WAN with MWCNT and PEDOT:PSS was carried out by adding 0.5 wt% MWCNT to the WAN dispersion, stirring for 30 min, and sonication for 10 min, followed by the addition of 0.5 wt% lyophilized PEDOT:PSS and 5 vol% DMSO. The mixture was then stirred for 30 min (350 rpm) and passed through a gauge 20 nozzle three times to facilitate shear mixing and get a uniform conductive ink. The ionically conductive amyloid nanofibril ink was prepared by adding 0.5 M KCl (Sigma‐Aldrich) to WAN‐Glut (pH 10.0, 2 wt%), followed by stirring for 30 min at 350 rpm.

For the preparation of electrically conductive (pH 10.0, 2 wt% WAN; 0.5 wt% MWCNT; 0.5 wt% PEDOT:PSS; 5 vol% DMSO) and ionically conductive (pH 10.0, 2 wt% WAN‐Glut; 0.5 M KCl) soft amyloid nanofibril‐based circuits, four tubular interfacial complex coacervates were extruded parallel to each other into LAN complexation bath (pH 2.0, 2 wt%, 20 vol% isopropyl alcohol), connecting two copper electrodes. The related test was performed using an M204 Autolab multichannel electrochemical workstation (Metrohm).

#### 4.1.5 Preparation of Viscoelastically Tunable and Glow‐in‐the‐Dark WAN Inks

To adjust the viscoelastic properties of the WAN (pH 10.0, 2 wt%), various concentrations of hydroxypropyl methylcellulose (HPMC) (1–5 wt%, Sigma‐Aldrich) were added to the WAN, followed by uniform dispersion using a homogenizer. The prepared mixture was processed in a vacuum chamber and then refrigerated for 24 h to remove trapped bubbles. The glow‐in‐the‐dark ink was prepared by adding various concentrations (1–10 wt%) of a persistent luminescent pigment (SrAl_2_O_4_:Eu^2+^, Dy^3+^, supplied by TechnoGlow with SKU: P02‐GRN‐M004Z, with particle size 50 µm according to the manufacturer) to the HPMC‐reinforced WAN ink (pH 10.0, 2 wt% WAN, 2 wt% HPMC), followed by thorough mixing to ensure uniformity. To improve dispersion and stability, the luminescent pigment was treated with (3‐aminopropyl)trimethoxysilane (APTMS, Sigma‐Aldrich) prior to addition to the HPMC‐reinforced WAN ink, thereby enhancing its interaction with WAN and stabilizing it in the ink. Accordingly, 2 g of the pigment was added to 50 mL of ethanol and stirred at 600 rpm for 10 min. While stirring, 5 vol% APTMS was added to the mixture and stirred for an additional 2 h. The resulting mixture was centrifuged at 6000 rpm and washed twice with ethanol and water to remove excess APTMS. The powder was dried at 90°C for 2 h and then added to the HPMC‐reinforced WAN ink after cooling to room temperature. The glow‐in‐the‐dark interfacial complex coacervates were prepared by controlled extrusion of the ink (pH 10.0, 2 wt% WAN; 2 wt% HPMC; 1 wt% APTMS‐decorated SrAl_2_O_4_:Eu^2+^, Dy^3+^) into the LAN complexation bath (pH 2.0, 2 wt%, 20 vol% isopropyl alcohol).

### 4.2 Characterization

The amyloid nanofibrils were assessed by AFM to analyze their morphology and overall dimensions. Accordingly, a 10 µL amyloid nanofibrils dispersion at the considered pH (2.0–12.0) with a concentration of ∼0.01 wt% was deposited on a freshly cleaved mica (Ted Pella, diameter of 10 mm), followed by 2 min incubation. The mica surface was gently rinsed with deionized water and dried with a gentle stream of air. AFM imaging was performed using a Bruker MultiMode 8 Scanning Probe Microscope in soft tapping mode. Bruker RTESPA‐150 cantilevers were used for AFM analysis, and the oscillation amplitude was set to ∼90% of the free oscillation amplitude near the cantilever's natural resonance frequency. Images were processed with NanoScope Analysis, and fiber lengths were measured with FiberApp.

The electrophoretic mobility of the amyloid nanofibrils was assessed for WAN and LAN (0.1 wt%) at pH 2.0, 4.0, 6.0, 8.0, 10.0, and 12.0 using NaOH and HCl solutions to adjust pH. For WAN‐Glut and WAN‐Geni, freshly made samples were analyzed without dilution. The samples were loaded into a disposable electrophoresis capillary cell (Malvern Panalytical; DTS1070) and analyzed using a Malvern Panalytical Zetasizer Nano ZS. The samples' electrophoretic mobility was measured at 40 V, with a 1 min equilibrium time prior to each assessment, and five measurements were taken to obtain the mean ± standard deviation.

Small‐angle X‐ray scattering (SAXS) analyses were performed using a Bruker AXS Micro instrument with a micro‐focused X‐ray source operating at 50 kV and 1000 µA. The Cu K_α_ X‐ray radiation source with *λ* = 1.5418 Å was collimated with a 2D Kratky collimator, and the data were collected with a 2D Pilatus 100K detector. The scattering vector *q*  =  (4π/*λ*) sin θ, with 2*θ* being the scattering angle, was calibrated using silver behenate. Data were collected and azimuthally averaged using the SAXSGUI software to yield 1D intensity versus scattering vector *q*, with a *q* range from 0.004 Å^−1^ to 0.5 Å^−1^. For all measurements, the samples were placed inside a quartz capillary with a diameter of ∼1.5 mm. Measurements were performed at 25°C, 80°C, and again after cooling to 25°C, while scattered intensity was collected over 5 min. Pair distance distribution function (PDDF) analysis was carried out using “primus” from ATSAS.

Fourier‐transform infrared (FTIR) spectroscopy of the samples was performed using a Nicolet S‐20 (Thermo Fisher Scientific). Morphological analyses of samples were carried out in dry or lyophilized form using a Tescan Mira 3 XMU field‐emission scanning electron microscope (FESEM) equipped with an energy‐dispersive spectrometer (EDS; Oxford Instruments). The dynamic and static contact angles and microscopic visualization of the formed proteinaceous interfacial complex coacervates were measured using the DSA KRÜSS Scientific (model DSA25E) pendant drop tensiometer. The electrical conductivity of the conductive thin films was analyzed via a four‐point probe Loresta GP resistivity meter (Mitsubishi Chemical Co., model MCP‐T700).

The fluorescence emissions of the proteinaceous interfacial complex coacervates or functionalized amyloid nanofibrils were analyzed with Leica Stellaris SP8‐DIVE or Zeiss LSM 880 Airyscan confocal microscopes using a 405 nm laser. The viscoelastic and rheological properties of the reinforced WAN (pH 10.0, 2 wt%) with various HPMC concentrations (1–5 wt%) were analyzed using an MCR 102 Anton‐Paar rotational rheometer, equipped with a parallel plate fixture with a diameter of 25 mm and a gap of 0.8 mm. Strain‐sweep tests (0.01%–1000%) at 1 Hz were performed to characterize the linear viscoelastic region (LVR). The flow characteristics of the samples were analyzed over the shear rate range of 0.01–1000 s^−1^, while the frequency sweep test was performed from 0.0628 rad/s to 628 rad/s at a strain of 1% within the LVR range. The elastic recovery of the samples was analyzed using dynamic oscillatory strain cycles at 1 Hz, with a low shear strain of 1% applied for 5 min, followed by a high shear strain of 300% for the next 5 min. All the analyses were performed under ambient conditions.

Temperature changes and thermal gradient maps were recorded using a FLIR E8 infrared camera. The photoluminescence (PL) intensity of the reinforced gel (WAN: pH 10.0, 2 wt%; HPMC: 2 wt%) with various concentrations of APTMS‐treated SrAl_2_O_4_:Eu^2+^, Dy^3+^ (1–10 wt%) was analyzed in a dark room under ambient conditions using a Canon EOS RP camera fixed on a tripod, equipped with a Canon Lens RF85mm F2 MACRO IS STM, with a focal length of 85 mm. The acquired images were analyzed using ImageJ to monitor changes in PL intensity over time within a fixed region of interest across all samples, consistent with recent studies [48].

Throughout the study, the pH of the suspensions was adjusted using METTLER TOLEDO or Thermo Scientific Orion Lab Star benchtop pH meters. The ionic conductivity was measured using VWR pHenomenal CO 3100L.

### 4.3 Electromagnetic Shielding Assessment

The electromagnetic interference (EMI) shielding of proteinaceous interfacial complex coacervates was performed using a Keysight (model P9374A) two‐port vector network analyzer (VNA) in the X‐band frequency range (8.2–12.4 GHz). The samples were positioned between the waveguide adaptors, and the complex scattering parameters S_11_, S_21_, S_22_, and S_12_ were measured to determine the shielding coefficients, including reflectance (*R*), transmittance (*T*), and absorbance (*A*), using the following formulations:

(1) $$R={\left|S_{11}\right|}^{2}={\left|S_{22}\right|}^{2}\;$$

(2) $$T={\left|S_{21}\right|}^{2}={\left|S_{12}\right|}^{2}\;$$

(3) A=1−R−T

Then, the calculated shielding coefficients are used to measure the reflection loss (SE*_R_*), absorption loss (SE*_A_*), and total shielding effectiveness (SE*_T_*) by the following formulations:

(4) $$SE_{R}=10\;\log\;\left(\frac{1}{1-R}\;\right)$$

(5) $$SE_{A}=10\;\log\left(\frac{1-R}{T}\right)$$

(6) $$SE_{T}=SE_{A}+SE_{R}$$

The mechanism of EMI shielding was defined as absorption‐dominant when *A* > 0.5 and reflection‐dominant when *A* < 0.5.

## Conflicts of Interest

The authors declare no conflicts of interest.

## Supporting information




**Supporting File 1**: adma74737‐sup‐0001‐SuppMat.docx.

**Supporting File 2**: adma74737‐sup‐0002‐VideoS1.mpg.

**Supporting File 3**: adma74737‐sup‐0003‐VideoS2.mpg.

**Supporting File 4**: adma74737‐sup‐0004‐VideoS3.mpg.

**Supporting File 5**: adma74737‐sup‐0005‐VideoS4.mpg.

**Supporting File 6**: adma74737‐sup‐0006‐VideoS5.mpg.

**Supporting File 7**: adma74737‐sup‐0007‐VideoS6.mpg.

**Supporting File 8**: adma74737‐sup‐0008‐VideoS7.mpg.

**Supporting File 9**: adma74737‐sup‐0009‐VideoS8.mpg.

**Supporting File 10**: adma74737‐sup‐0010‐VideoS9.mpg.

**Supporting File 11**: adma74737‐sup‐0011‐VideoS10.mpg.

**Supporting File 12**: adma74737‐sup‐0012‐VideoS11.mpg.

## Data Availability

The data that support the findings of this study are available from the corresponding author upon reasonable request.
