# Supplementary material for: All‐Amyloid Functional Coacervates
Source: Adv Mater. 2026 Aug 24;38(61):e74737. doi: 10.1002/adma.74737 (PMC13726710; doi:10.1002/adma.74737)
Supplement: Supplementary file 1 [file ADMA-38-e74737-s001.docx]

**Supporting Information**

**For**

**All-Amyloid Functional Coacervates**

Seyyed Alireza Hashemi^1,2^, Mohammad Peydayesh^2^, Laura Baraldi^2^, Mohammad Arjmand^1,*^, Raffaele Mezzenga^2,3,*^

^1^ Nanomaterials and Polymer Nanocomposites Laboratory, School of Engineering, University of British Columbia, Kelowna, BC, V1V 1V7, Canada

^2^ Department of Health Sciences and Technology, ETH Zürich, Zürich, 8092 Switzerland

^3^ Department of Materials, ETH Zürich, Wolfgang-Pauli-Strasse 10, 8049, Zürich, Switzerland

**Corresponding author(s):**

[mohammad.arjmand@ubc.ca](mailto:mohammad.arjmand@ubc.ca), [raffaele.mezzenga@hest.ethz.ch](mailto:raffaele.mezzenga@hest.ethz.ch)

**1. Supporting Figures and Related Explanations**

**Figure S1.** Birefringence of A) WAN and B) LAN (both at pH 2.0, 2 wt%) under cross-polarized light.

**Figure S2.** Functionalized WAN with various loadings of glutaraldehyde, from 1-15 wt%, considering the mass of WAN.

**Figure S3.** Functionalized WAN with various loadings of genipin, from 1-15 wt%, considering the mass of WAN.

**Figure S4.** Large AFM scan (60 µm × 60 µm) for WAN at pH 2.0 with a concentration of 0.01 wt%.

**Figure S5.** Large AFM scan (40 µm × 40 µm) for LAN at pH 2.0 with a concentration of 0.01 wt%.

**Figure S6.** Formation of all-amyloid interfacial complex coacervates upon extruding a droplet of WAN (pH 10.0, 2 wt%) into a LAN complexation bath (pH 2.0, 2 wt%), premixed with various volume ratios of isopropyl alcohol (0-40 vol%) before complexation.

**Figure S7.** Stability of formed all-amyloid interfacial complex coacervates upon extruding a droplet of WAN (pH 10.0, 2 wt%) into a LAN complexation bath (pH 2.0, 2 wt%, 20 vol% isopropyl alcohol), with and without heating (80 °C for 10 min).

**Figure S8.** Analyzing the pH-dependent complexation of amyloid nanofibrils when extruding WAN ink (2 wt%) with various pH values into the WAN complexation baths (2 wt%, 20 vol% isopropyl alcohol) with various pH values, followed by heating at 80 °C for 10 min, including baths with pH A) 2.0, B) 4.0, C) 6.0, D) 8.0, E) 10.0, and F) 12.0. The symbols denote the bath and ink pHs, respectively, e.g., W2W4: bath pH 2, ink pH 4.

**Figure S9.** Analyzing the pH-dependent complexation of amyloid nanofibrils when extruding LAN ink (2 wt%) with various pH values into the LAN complexation baths (2 wt%, 20 vol% isopropyl alcohol) with various pH values, followed by heating at 80 °C for 10 min, including baths with pH A) 2.0, B) 4.0, C) 6.0, D) 8.0, E) 10.0, and F) 12.0. The symbols denote the bath and ink pHs, respectively, e.g., L2L4: bath pH 2, ink pH 4.

**Figure S10.** Analyzing the pH-dependent complexation of amyloid nanofibrils when extruding WAN ink (2 wt%) with various pH values into the LAN complexation baths (2 wt%, 20 vol% isopropyl alcohol) with various pH values, followed by heating at 80 °C for 10 min, including baths with pH A) 2.0, B) 4.0, C) 6.0, D) 8.0, E) 10.0, and F) 12.0. The symbols denote the bath and ink pHs, respectively, e.g., L2W4: bath pH 2, ink pH 4.

**Figure S11.** The viscosity and gel-like behaviour of complexation baths at pH values of 4.0 and 6.0.

**Figure S12.** Effect of concentration on all-amyloid nanofibril complexation, when extruding WAN ink (pH 10.0) with various concentrations (0.1-2 wt%) into the LAN complexation bath (pH 2.0, 20 vol% isopropyl alcohol) with A) 0.1 wt%, B) 0.5 wt%, C) 1 wt%, D) 1.5 wt%, and E) 2 wt% concentrations.

**Figure S13.** Effect of concentration on all-amyloid nanofibril complexation, when extruding LAN ink (pH 2.0) with various concentrations (0.1-2 wt%) into the WAN complexation bath (pH 10.0, 20 vol% isopropyl alcohol) with A) 0.1 wt%, B) 0.5 wt%, C) 1 wt%, D) 1.5 wt%, and E) 2 wt% concentrations.

**Figure S14.** A-C) FESEM images of the skin formed around the capsules after freezing and lyophilization of the sample formed by extruding WAN ink (pH 10.0, 2 wt%) into the LAN complexation bath (pH 2.0, 2 wt%, 20 vol% isopropyl alcohol).

**Figure S15.** Raw SAXS data for A) WAN into WAN, B) LAN into LAN, and C) WAN into LAN systems before heating (25 °C), after heating at 80 °C, and after cooling to room temperature (25 °C).

**Figure S16.** FTIR analysis of the dried film of A) LAN (pH 2.0, 2 wt%), B) WAN (pH 2.0, 2 wt%), C) functionalized WAN with glutaraldehyde (pH 10.0, 2 wt%), and D) functionalized WAN with genipin (pH 10.0, 2 wt%).

**Explanation:** The FTIR spectra (Figure S16) display the characteristic vibrational bands of amyloid nanofibrils formed from *β*-lactoglobulin and lysozyme, as well as evidence of successful functionalization of WAN (*β*-lactoglobulin amyloid nanofibrils) with glutaraldehyde and genipin while preserving the amyloid backbone structure. The FTIR spectra of both LAN (Figure S16A; pH 2.0, 2 wt%) and WAN (Figure S16B; pH 2.0, 2 wt%) exhibit the characteristic protein bands corresponding to Amide III (1200-1400 cm^-1^), Amide II (1500-1600 cm^-1^), Amide I (1600-1700 cm^-1^), and N-H stretching vibrations (3060-3300 cm^-1^). The amide III band mainly arises from coupled N-H bending and C-N stretching vibrations of the peptide backbone, with smaller contributions from in-plane C=O bending and C-C stretching vibrations, and is associated with *β*-sheet structures. The amide II band originates from in-plane N-H bending and C-N stretching vibrations of the peptide backbone. The amide I band, as the strongest feature in amyloid nanofibrils' FTIR spectra, arises primarily from C=O stretching vibrations of the peptide bond and confirms the formation of a *β*-sheet-rich fibrillar structure characteristic of amyloid nanofibrils.^[1]^ WAN functionalization with glutaraldehyde led to clear changes in the resulting FTIR spectrum (Figure S16C; pH 10.0, 2 wt%), including an increase in the intensity of bands at 1400 cm^-1^ and 1450 cm^-1^, attributed to symmetric COO^-^ stretching vibrations and CH_2_ bending vibrations of protein side chains following glutaraldehyde functionalization; these changes combined with a slight shift and decrease in the intensity of the amide II band (from 1536 to 1540 cm^-1^) and the amide I band (from 1639 to 1643 cm^-1^) as a result of interaction with primary amines and the formation of Schiff-base crosslinking (imine linkage; C=N), confirm the successful functionalization with glutaraldehyde when compared with neat WAN.^[1a, 2]^ Furthermore, the preservation of the amide I band indicates that the *β*-sheet fibrillar structure of the amyloid nanofibrils remains intact after crosslinking. WAN functionalization with genipin led to clear changes in the resulting FTIR spectrum (Figure S16D; pH 10.0, 2 wt%), confirming successful modification. This includes a peak at 1081 cm^-1^, corresponding to in-plane C-H bending of genipin, and an increase in the intensities of bands at 1400 cm^-1^ and 1448 cm^-1^, attributed to symmetric COO^-^ stretching vibrations and CH_2_ bending vibrations of protein side chains associated with genipin-induced crosslinking. Additionally, a slight shift was observed in the amide II band (from 1536 to 1540 cm^-1^) and the amide I band (from 1639 to 1643 cm^-1^) as a result of interaction with primary amines and the formation of genipin-amine heterocyclic crosslinks, respectively.^[1a, 3]^

**Figure S17.** Raw SAXS data for A) WAN-Glut into LAN, B) WAN-Geni into LAN systems before heating (25 °C), after heating at 80 °C, and after cooling to room temperature (25 °C).

**Figure S18.** Fluorescence microscopy image of the WAN-Geni and LAN complexation at the liquid-liquid interface.

**Figure S19.** A-D) FESEM images of the skin formed around the capsules after freezing and lyophilization when extruding WAN-Glut ink (pH 10.0, 2 wt%) into the LAN complexation bath (pH 2.0, 2 wt%, 20 vol% isopropyl alcohol) at different magnifications or skin positions.

**Figure S20.** A-F) A view of the formed skin around the non-equilibrium-shaped tubular interfacial complex coacervate after freezing and lyophilization, when extruding WAN-Glut ink (pH 10.0, 2 wt%) into the LAN complexation bath (pH 2.0, 2 wt%, 20 vol% isopropyl alcohol), at different magnifications.

**Figure S21.** A-D) FESEM images of the skin formed around the capsules after freezing and lyophilization when extruding WAN-Geni ink (pH 10.0, 2 wt%) into the LAN complexation bath (pH 2.0, 2 wt%, 20 vol% isopropyl alcohol) at different magnifications or skin positions.

**Figure S22.** FTIR analysis of WAN (pH 10.0, 2 wt%) with various MWCNT concentrations: A) 0 wt%, B) 0.5 wt%, C) 1 wt%, D) 1.5 wt%, and E) 2 wt%.

**Explanation:** Figure S22 showcases the FTIR spectra of WAN (pH 10.0, 2 wt%; Figure S22A), reinforced with various MWCNT concentrations (0.5-2 wt%; Figures S22B-E). Accordingly, the WAN clearly shows the fingerprint FTIR spectrum of the amyloid nanofibril made from β-lactoglobulin, corresponding to Amide III (1200-1400 cm^-1^), Amide II (1500-1600 cm^-1^), Amide I (1600-1700 cm^-1^), and N-H stretching vibrations (~3264 cm^-1^).^[1a]^ Gradual addition of MWCNT from 0.5 wt% to 2 wt% led to a decrease in the intensity of peaks and their broadening. This could be due to the physical interaction between amyloid nanofibrils and MWCNTs, and to the broadband infrared attenuation caused by the graphitic structure in the MWCNTs, which partially shields the vibrational signals of the amyloid nanofibrils.

**Figure S23.** *i-ii*) FESEM images of WAN-MWCNT bicontinuous arrangements formed by samples with A) 1 wt%, B) 1.5 wt%, and C) 2 wt% MWCNT at different magnifications.

**Figure S24.** A) Formation of bicontinuous arrangements upon air-drying various WAN-MWCNT dispersions on glass substrates, and B) their respective contact angles at various MWCNT concentrations. C) Dynamic contact angle of air-dried WAN-MWCNT dispersions over time.

**Figure S25.** EDS analysis and elemental distribution map of bicontinuous arrangements formed via drying reinforced WAN (pH 10.0, 2 wt%) with A) 0.5 wt%, B) 1 wt%, C) 1.5 wt%, and D) 2 wt% concentrations of MWCNT on a glass substrate, where each part shows *i*) FESEM image of scanned area, *ii*) mixed FESEM with elemental distribution map, iii) carbon and oxygen elemental distribution map, and iv) EDS analysis.

**Figure S26.** A-F) FESEM images of the film formed via drying WAN (pH 10.0, 2 wt%) reinforced with 0.5 wt% MWCNT, 0.5 wt% PEDOT:PSS, and 5 vol% DMSO on a glass substrate at different magnifications.

**Figure S27.** EDS analysis and elemental distribution map of the film formed from drying WAN (pH 10.0, 2 wt%) reinforced with 0.5 wt% MWCNT, 0.5 wt% PEDOT:PSS, and 5 vol% DMSO, including A) FESEM image of scanned area, B) mixed FESEM with elemental distribution map, C) carbon and oxygen elemental distribution map, and D) EDS analysis.

**Figure S28.** A-C) FESEM images of the thin film formed by drying the WAN ink (pH 10.0, 2 wt%) on a glass substrate at different magnifications.

**Figure S29.** A-F) FESEM images of the film formed via drying WAN (pH 10.0, 2 wt%) reinforced with 0.5 wt% PEDOT:PSS and 5 vol% DMSO on a glass substrate at different magnifications.

**Figure S30.** EDS analysis and elemental distribution map of the film formed from drying WAN (pH 10.0, 2 wt%) reinforced with 0.5 wt% PEDOT:PSS and 5 vol% DMSO, including A) FESEM image of scanned area, B) mixed FESEM with elemental distribution map, C) carbon and oxygen elemental distribution map, and D) EDS analysis.

**Figure S31.** A-F) FESEM images of the 0.5 wt% MWCNT interfacial complex coacervate film, formed by casting a LAN complexation bath (pH 2.0, 2 wt%, 20 vol% isopropyl alcohol) on a pre-cast WAN-MWCNT ink on a glass substrate (WAN: pH 10.0, 2 wt%; MWCNT: 0.5 wt%) at different magnifications.

**Figure S32.** EDS analysis and elemental distribution map of the 0.5 wt% MWCNT interfacial complex coacervate film, formed by casting the LAN complexation bath (pH 2.0, 2 wt%, 20 vol% isopropyl alcohol) on a pre-cast WAN-MWCNT ink on a glass substrate (WAN: pH 10.0, 2 wt%; MWCNT: 0.5 wt%), including A) FESEM image of scanned area, B) mixed FESEM with elemental distribution map, C) carbon and oxygen elemental distribution map, and D) EDS analysis.

**Figure S33.** A-L) FESEM images of the 0.5 wt% MWCNT-0.5 wt% PEDOT:PSS interfacial complex coacervate film, formed by casting the LAN complexation bath (pH 2.0, 2 wt%, 20 vol% isopropyl alcohol) on a pre-cast WAN-MWCNT-PEDOT:PSS ink on a glass substrate (WAN: pH 10.0, 2 wt%; MWCNT: 0.5 wt%; PEDOT:PSS: 0.5 wt% + 5 vol% DMSO) at different magnifications, showing the formation of wrinkles all around the formed layer upon the complexation of oppositely charged amyloid nanofibrils.

**Figure S34**. EDS analysis and elemental distribution map of the 0.5 wt% MWCNT-0.5 wt% PEDOT:PSS interfacial complex coacervate film, formed by casting the LAN complexation bath (pH 2.0, 2 wt%, 20 vol% isopropyl alcohol) on a pre-cast WAN-MWCNT-PEDOT:PSS ink on a glass substrate (WAN: pH 10.0, 2 wt%; MWCNT: 0.5 wt%; PEDOT:PSS: 0.5 wt% + 5 vol% DMSO), including A) FESEM image of scanned area, B) mixed FESEM with elemental distribution map, C) carbon and oxygen elemental distribution map, and D) EDS analysis.

**Figure S35.** A) Ionically conductive liquid wires formed via extruding WAN-Glut (pH 10.0, 2 wt%, 0.5 M KCl) in the LAN complexation bath, and B) their ability to light up an LED at an applied voltage of 3 V.

**Figure S36.** Stable current and voltage responses over time for the ionically conductive liquid wires formed via extruding WAN-Glut (pH 10.0, 2 wt%, 0.5 M KCl) in the LAN complexation bath.

**Figure S37.** A) Formation of ionically conductive WAN-Glut interfacial complex coacervates in a LAN complexation bath, and shielding parameters of an 8 mm thick sample (3 cm diameter), including B) shielding coefficients, C) EMI SE parameters, and D) EMI SE over frequency (X-band frequency range, 8.2-12.4 GHz).

**Figure S38.** The strain sweep test of WAN (pH 10.0, 2 wt%) with A) 0 wt%, B) 1 wt%, C) 2 wt%, D) 3 wt%, E) 4 wt%, and F) 5 wt% HPMC.

**Figure S39.** The elastic recovery test of WAN (pH 10.0, 2 wt%) with A) 0 wt%, B) 1 wt%, C) 2 wt%, D) 3 wt%, E) 4 wt%, and F) 5 wt% HPMC.

**2. Details of Supporting Videos:**

**Video S1:** Forming a non-equilibrium-shaped proteinaceous interfacial complex coacervate via extruding WAN ink (pH 2.0, 2 wt%) into the WAN complexation bath (pH 8.0, 2 wt%, 20 vol% isopropyl alcohol).

**Video S2:** Forming a non-equilibrium-shaped proteinaceous interfacial complex coacervate via extruding LAN ink (pH 12.0, 2 wt%) into the LAN complexation bath (pH 2.0, 2 wt%, 20 vol% isopropyl alcohol).

**Video S3:** Forming a non-equilibrium-shaped proteinaceous interfacial complex coacervate via extruding WAN ink (pH 10.0, 2 wt%) into the LAN complexation bath (pH 2.0, 2 wt%, 20 vol% isopropyl alcohol).

**Video S4:** Formation of wrinkles on a proteinaceous interfacial complex coacervate with pendant drop-shaped sections via extruding WAN ink (pH 10.0, 2 wt%) into the LAN complexation bath (pH 2.0, 2 wt%, 20 vol% isopropyl alcohol).

**Video S5:** Controlling the features and the flexible nature of the proteinaceous interfacial complex coacervate formed via extruding WAN ink (pH 10.0, 2 wt%) into the LAN complexation bath (pH 2.0, 2 wt%, 20 vol% isopropyl alcohol).

**Video S6:** Forming a non-equilibrium-shaped proteinaceous interfacial complex coacervate via extruding glutaraldehyde-functionalized WAN (WAN-Glut) into the LAN complexation bath (pH 2.0, 2 wt%, 20 vol% isopropyl alcohol).

**Video S7:** Forming a non-equilibrium-shaped proteinaceous interfacial complex coacervate via extruding genipin-functionalized WAN (WAN-Geni) into the LAN complexation bath (pH 2.0, 2 wt%, 20 vol% isopropyl alcohol).

**Video S8:** A view of an initially high contact angle, followed by water absorption via a bicontinuous arrangement formed by WAN-MWCNT (WAN: pH 10.0, 2 wt%, MWCNT: 1.5 wt%).

**Video S9:** Photoluminescence intensity over time for WAN-HPMC (WAN: pH 10.0, 2 wt%; HPMC: 2 wt%) gels reinforced with various concentrations of APTMS-SrAl_2_O_4_:Eu^2+^, Dy^3+^ (1-10 wt%) compared with pure APTMS-SrAl_2_O_4_:Eu^2+^, Dy^3+^ powder.

**Video S10:** Developing a proteinaceous luminescence lamp by placing a 10 mL glass vial containing WAN-HPMC (WAN: pH 10.0, 2 wt%; HPMC: 2 wt%) gel reinforced with 10 wt% APTMS-SrAl_2_O_4_:Eu^2+^, Dy^3+^ in a diffusive reflector.

**Video S11:** Glow-in-the-dark interfacial complex coacervate via extruding WAN-HPMC (WAN: pH 10.0, 2 wt%; HPMC: 2 wt%) ink reinforced with 1 wt% APTMS-SrAl_2_O_4_:Eu^2+^, Dy^3+^ into the LAN complexation bath (pH 2.0, 2 wt%, 20 vol% isopropyl alcohol).

**References:**

[1]	a)A. Barth, *Biochimica et Biophysica Acta (BBA)-Bioenergetics* **2007**, 1767, 1073; b)G. Zandomeneghi, M. R. Krebs, M. G. McCammon, M. Fändrich, *Protein Science* **2004**, 13, 3314; c)M. Peydayesh, S. Kistler, J. Zhou, V. Lutz-Bueno, F. D. Victorelli, A. B. Meneguin, L. Spósito, T. M. Bauab, M. Chorilli, R. Mezzenga, *Nature Communications* **2023**, 14, 1848.

[2]	a)S. Farris, J. Song, Q. Huang, *Journal of Agricultural and Food Chemistry* **2010**, 58, 998; b)R. Rodrigues de Melo, R. C. Alnoch, A. F. L. Vilela, E. Maltempi de Souza, N. Krieger, R. Ruller, H. Harumi Sato, C. Mateo, *Molecules* **2017**, 22, 1088.

[3]	H. Khan, R. Shukla, A. Bajpai, *Materials Science and Engineering: C* **2016**, 61, 457.
